# What is the impact of intellectual property rules on access to medicines? A systematic review

**DOI:** 10.1186/s12992-022-00826-4

**Published:** 2022-04-15

**Authors:** Brigitte Tenni, Hazel V. J. Moir, Belinda Townsend, Burcu Kilic, Anne-Maree Farrell, Tessa Keegel, Deborah Gleeson

**Affiliations:** 1 School of Psychology and Public Health, La Trobe University, Plenty Rd &, Kingsbury Dr, Bundoora, VIC 3086 Australia; 2 Nossal Institute for Global Health, University of Melbourne, 5th Floor, 333 Exhibition Street, Melbourne, VIC 3000 Australia; 3 College of Arts and Social Sciences, The Australian National University, Canberra, Australia; 4 Menzies Centre for Health Governance, The Australian National University, Canberra, Australia; 5 Digital Rights Program, Public Citizen, Washington D.C, USA; 6 Edinburgh Law School, University of Edinburgh, Edinburgh, UK; 7 Monash Centre for Occupational and Environmental Health, School of Public Health and Preventive Medicine, Monash University, Melbourne, Australia

**Keywords:** Intellectual property, Patents, Generic medicines, Data exclusivity, Compulsory licencing, TRIPS flexibilities, Trade agreements, TRIPS-plus

## Abstract

**Background:**

It is widely accepted that intellectual property legal requirements such as patents and data exclusivity can affect access to medicines, but to date there has not been a comprehensive review of the empirical evidence on this topic. The World Trade Organization’s Agreement on Trade-Related Aspects of Intellectual Property Rights (TRIPS) requires Member States to implement minimum standards of intellectual property protection including patents for pharmaceutical products, but also contains ‘flexibilities’ designed to address barriers to access to medicines. National intellectual property laws can also include TRIPS-plus rules that go beyond what is required by TRIPS. We aimed to systematically review literature that measures the impact of intellectual property rules on access to medicines, whether implemented as a result of TRIPS, TRIPS-plus provisions in other trade agreements, or unilateral policy decisions.

**Methods:**

We searched Proquest, SCOPUS, Web of Science, PubMed, JSTOR, Westlaw and Lexis Nexis. Peer reviewed articles, government reports and other grey literature were included. Articles were eligible for inclusion if they were quantitative, in English, included a measure of cost, price, availability of or access to medicines, were about intellectual property or data exclusivity rules and published between January 1995 and October 2020. Ninety-one studies met our inclusion criteria. We systematically reviewed the studies’ findings and evaluated their quality using a modified quality assessment template.

**Results and conclusion:**

Five broad overarching themes and 11 subthemes were identified based on the articles’ foci. They were: trade agreements (divided into EU FTAs and those that include the USA); use of TRIPS flexibilities (divided into compulsory licencing and parallel importation); patent expiry/generic entry/generic pathway (divided into comparative studies and single country studies); patent policies (also divided into comparative studies and single country studies) and TRIPS-plus rules (divided into data exclusivity, patent term extensions and secondary patenting). Most studies focused not on specific trade agreements, but on TRIPS-plus provisions, which can also be found within some trade agreements.

The main finding of this review is that the stronger pharmaceutical monopolies created by TRIPs-plus intellectual property rules are generally associated with increased drug prices, delayed availability and increased costs to consumers and governments. There is evidence that TRIPS flexibilities can facilitate access to medicines although their use is limited to date. There were few studies that included resource poor settings, signalling a need for greater research in such settings where the impact on access to medicines is likely to be more damaging.

**Supplementary Information:**

The online version contains supplementary material available at 10.1186/s12992-022-00826-4.

## Background

The Agreement on Trade-Related Aspects of Intellectual Property Rights (TRIPS) is one of the core World Trade Organization (WTO) agreements and came into effect in 1995 [1]. It includes minimum legal standards of intellectual property (IP) protection including patents for pharmaceutical products. All WTO Member States must comply with TRIPS by implementing its requirements in their national IP laws. TRIPS also contains flexibilities designed to respond to concerns that patents and monopoly pricing are barriers to access^1^ to medicines [2, 3]. These flexibilities include but are not limited to compulsory licencing, Bolar provisions, limits to the scope of patentability, definitions of invention, parallel importation and the least developed country (LDC) transition period. See table below.

**Table:** 

**Definitions for some TRIPS flexibilities**	
**terms**	**meaning**
Compulsory licencing	Compulsory licencing is the authorisation of the production of a patented product or use of a patented process without the consent of the patent owner. The compulsory licensee must pay a royalty fee to the patent holder [4].
Bolar/early working provision	A “Bolar/early working” provision allows generic drug manufacturers to use the patented invention to obtain marketing approval without the patent owner’s permission so that the generic product is approved to enter the market as soon as the patent expires [5]. It is within the scope of ‘Article 30 Exemption to Rights Conferred’ of the TRIPS Agreement [6].
Parallel importation	Parallel importation is when a country imports an authorised patented product, (presumably at a cheaper price) from another country without the permission of the patent owner [2]. This is effectively international exhaustion of patent rights, allowed for by TRIPS Article 6 [6].
LDC transition period	The LDC transition period frees LDCs from TRIPS obligations related to patents on pharmaceuticals until 2033 or until they are no longer a LDC [7]. This allows LDCs to purchase and/or produce cheaper generic medicines.

Many countries, especially low- and middle-income countries (LMICs) were concerned that having to grant patents for medicines would increase prices and compromise governments’ ability to protect public health. The 2001 WTO Doha Declaration on the TRIPS Agreement and Public Health (Doha Declaration) reaffirms Member States’ rights to make full use of flexibilities within the TRIPS Agreement in order to protect public health and maximise access to medicines [8].

IP law can also be influenced by trade agreements negotiated outside the WTO. In recent years, some countries, especially those that are net exporters of IP such as the USA, Japan and the European Union (EU), have pursued bilateral and/or plurilateral trade agreements that include IP rules that go beyond those required by TRIPS. These TRIPS-plus rules can include extending patent terms beyond 20 years, expanding patentability, limiting the grant of compulsory licences and extending data exclusivity [9–12]. Not all national IP laws, however, are the consequence of trade agreements. Many countries have unilaterally introduced TRIPS-plus IP laws independent of trade agreements, some prior to the TRIPS Agreement.

Over the years, researchers in various fields of study including, law, public health and economics have aimed to quantify the impact of IP rules such as strengthened patent protection, data exclusivity and patent term extension on the price and availability of medicines. Data exclusivity refers to the protection of clinical drug trial data submitted to drug regulatory authorities for market approval. It operates to prohibit generic drug companies from relying on existing drug trial data submitted to regulatory authorities to obtain market approval for a generic medicine [13, 14].

To date, there have been systematic reviews that have summarised the evidence regarding the health impact of trade agreements [15], the impacts of IP provisions in trade agreements on access to medicine in LMICs [16] and the impact of specific plurilateral trade agreements on the accessibility and affordability of medicines [17]. Findings from these reviews suggest trade agreements pose significant health risks [15], IP protection in trade agreements increases medicine prices and decreases consumer welfare in LMICSs [16] and the Trans-Pacific Partnership Agreement (TTPA) could hinder affordability and accessibility to medicines [17]. No systematic review, however, has captured the full range of empirical studies that have focused on the impact of IP rules on access to medicines globally.

The aim of this study is to systematically review the literature that measures the impact of IP rules on access to medicines. This evidence can be used to inform relevant trade and IP law and policy development to enhance access to affordable medicines or at least minimise negative effects. We defined ‘impact’ as the availability, cost or price^2^ of medicines. IP rules relevant to access to medicines include, but are not limited to, pharmaceutical product patents, data exclusivity, secondary patenting, patent term extension, patent linkage, along with TRIPS flexibilities including compulsory licencing, Bolar provisions and parallel importation. Patent linkage links marketing approval by the drug regulator to the patent status of the drug [18]. A secondary patent is a patent on aspects other than the original active drug ingredient, such as chemical variants, new formulations of the drug or methods of administration [19]. It is used to extend the effective patent life of a drug.

## Methods

### Search strategy

The search strategy for the review was developed with guidance from a librarian who is an expert on systematic reviews. A scoping exercise was first undertaken to develop and revise the search terms. The review protocol was registered in the PROSPERO database (registration number CRD42018106579).

In January 2018 we searched the following databases: Proquest, SCOPUS, Web of Science, PubMed, JSTOR, Westlaw and Lexis Nexis using the following search string:

**Table:** 

For intellectual property rules	TRIPS OR “intellectual property” OR IP OR patent* OR evergreening OR “patent term” OR “compulsory licens*” OR (“parallel import*” NOT books) OR (“data protection” NOT privacy) OR “data exclusivity” OR “patent linkage” OR “marketing approval” OR “regulatory approval” OR Bolar
medicines	medicine* OR pharamaceut*
Impact	NEAR/5 (access* OR availabl* OR cost OR price

Together these databases cover many thousands of journals containing millions of records from the disciplines of life sciences, biomedical sciences, medicine, engineering, social sciences, arts and humanities, politics, international relations and law.

This search was repeated with the same databases in October 2020 to include literature published from January 2018 to October 2020. The reference lists of the included articles were also searched for relevant literature and any relevant articles that had not been captured by the search, were added manually for screening. An expert panel of three academics experienced in empirical research and widely published in the field of IP and access to medicines, reviewed our list of included articles and suggested possible additions. These articles were scanned and those that met the inclusion criteria were added. The literature included peer reviewed articles, government reports and other grey literature.

### Screening and selection criteria

After duplicates were removed, a total of 3004 articles were screened by a team of four, using the systematic review management tool, Covidence (Covidence systematic review software, www.covidence.org). Titles and abstracts were independently screened by two reviewers. Three hundred and seven articles were assessed at the full text stage for eligibility, again by two reviewers. These were assessed against the following inclusion criteria:

i) quantitative
ii) in English
iii) included a measure of cost, price, availability of or access to medicines
iv) about intellectual property or data exclusivity rules
v) published between January 1995 and October 2020.

**Fig. 1:**
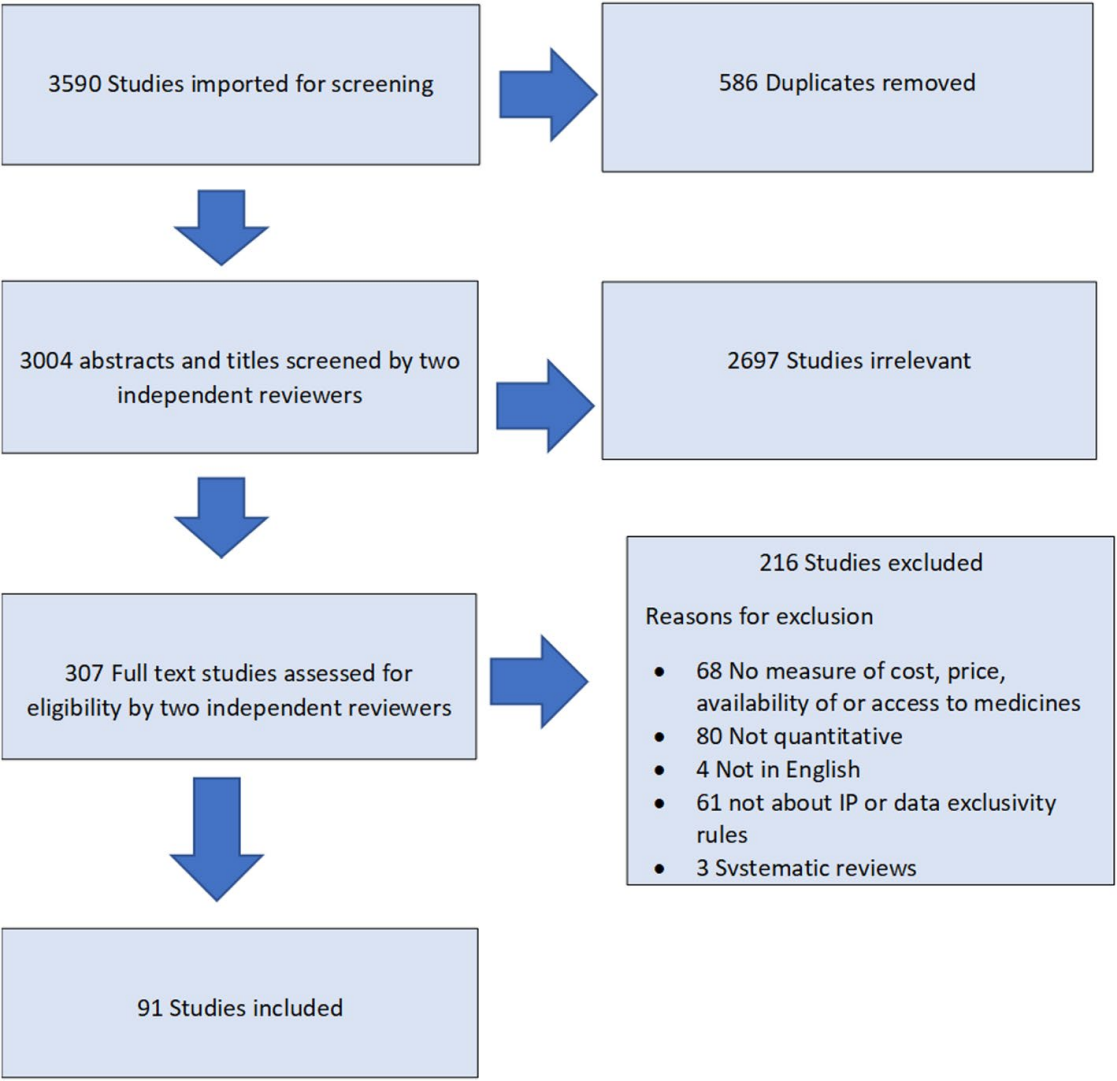
PRISMA chart

Systematic reviews were excluded from our review. However, we reviewed all articles included in any systematic reviews that were retrieved in our search and added any studies that met our inclusion criteria. Any conflicts in screening were discussed and resolved as a group. Ninety-one articles from 1995 to 2020 met our inclusion criteria for synthesis.^3^ See Fig. 1 for a PRISMA chart of the screening and review process [20].

### Data extraction

Four of the authors undertook the data extraction using a template based on knowledge of the field and of systematic review approaches. This included: publication date; years studied; geographic focus; research question/ objective; study design; study data; IP rule(s) studied; outcome measure(s); method of analysis; main findings; and funding source. See supplementary file 1 for the data extraction template. Two authors independently completed data extraction forms for each full-text article included in the review and the lead author compiled a composite version at the end of the extraction.

### Quality assessment

The team developed a quality assessment template informed by best practice guidance [21] and appraised the included 91 articles on a sliding scale based on the strengths of the following: Abstract and title; Introduction and aims; Method and data; Sampling; Data analysis; Ethics and bias; Findings/results; Transferability/ generalisability; and Implications and usefulness. These aspects were assessed independently and not given an aggregate score.

See supplementary file 2 for the quality assessment tool. Two authors independently completed quality assessment forms for each full-text article included. All articles with econometric or model-based analysis were also reviewed by the economist in the team. Any differences in findings were discussed and an agreement reached in terms of the strengths and quality of the article.

### Data analysis

No meta-analysis or meaningful quantitative analysis was possible given the heterogeneity of studies, their aims, methods and findings. Instead, a qualitative analysis was employed to collate articles into common themes. Examining the data extraction sheets for the 91 studies, the author team collaboratively identified these themes based on the article’s dominant focus, e.g. whether it examined a specific trade agreement, or an IP rule in a country or group of countries, and its impact on availability, cost, or price of medicines.

## Results

The 91 articles consisted of: 68 journal articles, 15 institutional reports/working papers, five government studies/reports and three PhD theses. The author team divided these articles into in five broad overarching themes and 11 subthemes described below.

### Trade agreements

Eighteen studies examined trade agreements. Articles under this theme were subdivided into trade agreements that include the EU and those that include the USA, as these are main parties demanding extensions to IP policy. One study examined both. See Table 1 below.

**Table 1:** Studies of trade agreements

Theme	Author(s), date and reference number	Study Type^**a**^/Methodology	Key relevant findings
**EU trade agreements**	**The Comprehensive Economic and Trade Agreement (CETA) (Canada)**		
	Grootendorst and Hollis 2011 [22]	Prospective modelling to determine how proposed provisions in CETA would affect public and private health care costs	CETA’s TRIPS-plus IP provisions would delay the market entry of generics and increase Canada’s annual pharmaceutical expenditure. Payers – consumers, businesses, unions and government insurers – would face substantially higher drug costs as exclusivity is extended on top-selling prescription drugs, with the annual increase in costs likely to be in the range of $C2.8 billion per year.
	Lexchin 2014 [23]	Prospective modelling study to determine the impact of the 3 settings in CETA - patent term restoration, data protection, rights of appeal on the cost of prescription drugs in Canada	Using 2010 data, the CETA data exclusivity provisions would have increased the average duration of market exclusivity for patented drugs by 358.4 days, or 0.98 years, which would bring an additional yearly cost of $C795 million, or 6.2% of the total annual cost of patented drugs. If data exclusivity were extended to non-innovative drugs, the average delay would increase by 741 days, or 2.03 years, which represents an additional yearly cost of $C1,645 million, or 12.9% of total costs of patented drugs.
	Beall et al.^a^ 2019 [24]	Prospective modelling study to evaluate the total combined impact of prolonging market protection by extending competition-blocking patent and data exclusivity terms implicit in the Canada, United States, and Mexico Trade Agreement and the Comprehensive Economic and Trade Agreement with the European Union.	The collective impact of these policy changes would be to extend the regulatory protection period for new drugs from an average of 10.0 years to 11.1 years. An 11% increase equates to an average of $C410 million annually (with a minimum estimate of $C40 million and a maximum of $C1.4 billion).
	**The EU-Columbia, Ecuador, Peru Trade Agreement (EU-Andean FTA)**		
	IFARMA HAI EU 2009- Andean FTA (Peru) [22]	Prospective scenario planning used to evaluate the foreseen impact on access to medicines in Peru from the IP measures (data exclusivity and patent term extensions through Supplementary Protection Certificates (SPCs)) proposed by the European Union as part of the EU- Andean FTA.	Estimates that these two measures would increase Peru’s total pharmaceutical expenditure in 2025 by $US 459 million if current consumption is maintained. Consumption decrease would be caused by the result of an 11% increase in the number of API protected, which in turn would lead to a 26% price increase. Implementing Article 9.3 SPC measures (thus extending the effective patent period by 4 years), would lead to a $US 159 million increase in pharmaceutical expenditure in 2025. At the same time, implementing the proposed 10-year test data exclusivity period would lead to an increase of more than $US 300 million USD in 2025 expenditure.
	IFARMA/HAI EU 2009- Andean FTA (Columbia) [23]	Prospective scenario planning used to evaluate the foreseen impact on access to medicines in Colombia from the IP measures proposed by the European Union as part of the EU-Andean FTA. Specifically assesses the impact of increasing the effective duration of pharmaceutical patents and test data protection.	It is estimated that the introduction of the two measures on data exclusivity and SPC would lead to an increase of $US756 million in Colombia’s total pharmaceutical expenditure in 2025, and at the same time, a decrease in consumption of 10%. The consumption decrease is caused by an 8% increase in the number of IPR protected products, which in turn would produce a 16% price increase.
**US trade agreements**	**Thailand-US Trade Agreement**		
	Yoongthong 2015 [25]	Prospective modelling study to estimating impact of TRIPS-plus measures on the Thailand economy through consumer welfare estimate.	The enforcement of proposed TRIPS-plus would result in a substantial loss to the Thai economy, ranging between 30 billion baht and 136 billion baht, within a 10-year period from 2012 to 2021.
	Kessomboon et al. 2010 [26]	Prospective scenario modelling of the impact of the TRIPS-plus provisions set out by the Thai-US FTA proposal – a 10-year patent term extension, 10-year data exclusivity and patent linkage leading to a 5-year delay in generic entry. It used the Model of Impact of Changes in Intellectual Property Rights (MICIPR) framework.	The findings for all scenarios demonstrated a negative impact on the pharmaceutical market, particularly increasing drug expenditures, reducing access to medicines, and shrinking the domestic pharmaceutical industry. Comparison of the single provisions of the US proposal revealed the 10-year patent extension scenario would have the greatest negative impact: a 32% increase in the price index for medicines; increased spending on medicines to approximately US$11,191 million; the domestic industry would lose US$3370 million. The impact of all three provisions combined, over the next 20 years (2027) would be: 67% of medicine prices would increase: pharmaceutical expenditures would increase to US$23,595 million; the domestic industry could lose US$9000 million.
	Akaleephan et al. 2009 [27]	Prospective modelling study to estimate the impact of market exclusivity extension on the price of medicines under the proposed TRIPS-plus US-FTA model and to estimate the current potential cost saving loss and accessibility to medicines.	In 2003, the availability of generics would help to save 104.5% of actual expense and accessibility would increase by 53.6%. The extension of market exclusivity was estimated to lead to a cumulative potential expense of $US 6.2 million for the first year rising to $US 5215.8 million in the tenth year.
	**US-Jordan Trade Agreement**		
	Malpani 2009 [28]	Retrospective analysis of the impact of TRIPS-plus measures on affordability and availability of medicines in Jordan. Evaluates the claims of the benefits of TRIPS-plus rules for Jordan.	Data exclusivity prevents generic competition for 79% of medicines launched by 21 multinational pharmaceutical companies between 2001 and 2006. Additional expenditures for medicines with no generic competitor, as a result of enforcement of data exclusivity, were between $US6.3 m and $US22.04 m.
	Abbott et al. 2019 [26]	Retrospective analysis of the impact of Jordan’s increased IP protection, as a result of WTO accession and the US-Jordan FTA, on access to medicines. Quantifies the impact from delaying the entry of generics due to IP protections on the private retail market.	When assessing originator medicines that were marketed in both 1999 and 2004, and for which there were generic equivalents, the weighted average price of originator medicines increased while the weighted average price of equivalent generic medicines decreased. Delayed market entry of generics due to enhanced IP protection is estimated to have cost Jordanian private consumers approximately $US18 million in 2004. After adjusting for increased sales volume and inflation, from 1999 to 2004 there was a 17% increase in total annual expenditure for medicines in Jordan.
	**US-Korea Trade Agreement (KORUS)**		
	Oh and Kim 2012 [29]	Prospective study to estimate the impact of IP measures in the KORUS trade agreement on market size and welfare on the Korean economy.	When KORUS is finalized, estimated producers’ annual sales loss and consumers’ annual loss will be 0.223 and 0.155% of the total market size, respectively, due to patent extensions compensating for delayed patent registration. In the case of linking original patents to generic drug approval, producers’ annual sales loss and consumers’ annual loss will be 0.789 and 0.546% of total market size, respectively. When the market size of original drugs and generics is assumed to be $US3.0 billion, total welfare loss (the sum of producer’s loss and consumer’s loss) will be $US11.34 million due to delayed registration and $US 40.03 million due to patent linkage.
	Son 2020 [30]	A prospective statistical modelling study to capture the availability of new medicines, measure drug lags for new medicines, and to demonstrate the effect of the KORUS FTA on the timely availability of new medicines in the Korean market.	The KORUS FTA does not increase the availability of new medicines or shorten the drug lag of new medicines. However, the presence of the manufacturer in Korea was significantly related to the availability and drug lag in the Korean market. It is noteworthy that the presence of the manufacturer, which is a kind of by-product of free trade in pharmaceuticals, affected drug lag.
	**Canada-US-Mexico Agreement (CUSMA) (Canada)**		
	Beall et al.^a^ 2019 [24]	Prospective modelling study to evaluate the total combined impact of prolonged periods of market protection by extending competition-blocking patent and data exclusivity terms implicit in the Canada, United States, and Mexico Trade Agreement and the Comprehensive Economic and Trade Agreement with the European Union.	The collective impact of these policy changes will be to extend the regulatory protection period for new drugs from an average of 10.0 years to 11.1 years. An 11% increase equated to an average of $US 410 million annually (with a minimum estimate of $US 40 million and a maximum of $US 1.4 billion).
	Lexchin 2019 [31]	Prospective statistical modelling to investigate the effect the renegotiated North American Free Trade Agreement’s (NAFTA) increase in data protection for biologics from 8 to 10 years on drug spending in Canada.	Depending on how much of the market is captured by biologic competitors and how strong the patents are, lost savings from data protection extension could range from $US 0 to $US 305.8 million. One biologic competitor currently on the market could theoretically have been affected by an increase in data protection. Increased data protection would have had minor effects on products that have already lost data protection.
	Parliamentary Budget Office 2019 [32]	Prospective modelling to estimate the potential cost of the extended term of data protection. It defines that cost as the additional expenditures on originator prescription biologics relative to their potential competitors.	In 2015, some 16 biologics worth $US 422.4 million in prescription sales had data protection expiring between 2015 and 2023. By 2023, all drugs with data protection in 2015 would have lost it, were it not for CUSMA. On average, $US52.8 million worth of sales would have lost data protection annually over that period ($US422.4 million divided by eight years). Effectively, for those drugs whose primary patent expires before the extended data protection, CUSMA would have delayed the entry of lower cost biosimilars that would have competed for market share. PBO assumed that the discount from a biosimilar would be 30% and that biosimilars would affect sales in 75% of the market of the biologics losing data protection. As a result of the delay, the annual average increase in drug costs would amount to $US11.9 million per year. Doubling this number to account for the fact that it is a two-year extension produces an annual average increase in costs of $US23.8 million between 2015 and 2023.
	**The Central America Free Trade Agreement (CAFTA) (Guatemala)**		
	Shaffer and Brenner 2009 [33]	Retrospective study of effect of data exclusivity in CAFTA on access to medicines in Guatemala	CAFTA’s data exclusivity and patent rules as implemented in Guatemala through domestic law and regulation are limiting access to some more affordable generic drugs. Some drugs would become open for generic competition in the US (where they were first launched) before Guatemala. Some generic competitors denied entry to market, or removed from market, in Guatemala.
	**The US-Chile Free Trade Agreement (FTA)**		
	Trachtenberg et al. 2020 [34]	Retrospective study to measure the strength of IP provisions in Chile’s FTAs and to examine the extent to which FTAs with strong IP provisions impact the volume, unit value and overall value of imported biologic medicines into Chile.	FTAs with more stringent IP provisions increase both the volume and the unit value of imported biologics, suggesting greater availability of imported biologics at a higher price.
	**The Trans-Pacific Partnership (TPP) (Vietnam)**		
	Moir et al. 2018 [35]	Prospective modelling to estimate the potential impact of the TPP on cost and thus access to HIV treatment in Vietnam.	At current funding levels 82% of Vietnam’s eligible people living with HIV would receive ARVs if TRIPS legal flexibilities were fully utilised, while as few as 30% may have access to ARVs under the TPP Agreement – more than halving the proportion currently treated.
	**All US Trade Agreements**		
	Bollyky 2016 [36]	Prospective modelling study to determine whether trade deals with the US have increased medicine prices, spending on medicines or caused a shift away from lower cost generics.	For countries with trade deals with the USA: national pharmaceutical spending as a share of total health expenditure has not increased; growth in per capita pharmaceutical spending has not increased in comparison with comparator countries; consumption of pharmaceuticals has not increased; proportions of branded vs generic drugs consumed has not increased; and average price of off-patent originator medicines launched before and after US trade deals has not increased. The study does not take into account that many of the TRIPS-plus provisions in the trade agreements did not come into effect during the period studied.

^a^Explores both CETA and CUSMA/USMCA/T-MEC

#### EU FTAs

Of the five articles about EU treaties, three were prospective^4^ modelling studies that explore the impact of the EU-Canada Comprehensive Economic and Trade Agreement (CETA) on Canada’s public and private health care costs [22] and prescription drug prices [23, 24]. These CETA studies found that CETA’s TRIPS-plus IP provisions would delay the market entry of generics and increase Canada’s annual pharmaceutical expenditure [22–24]. The other two EU FTA articles were prospective analyses of the impact of the EU-Andean Agreement’s IP provisions on access to medicines in Peru [37] and Columbia [38]. These two studies found that the introduction of data exclusivity and patent term extensions (Supplementary Protection Certificates (SPCs))^5^ would increase total pharmaceutical expenditure and decrease consumption due to an increase in the number of active pharmaceutical ingredients under patent in Peru [37] and Columbia [38].

#### FTAs that include the USA

Articles that focused on US FTAs covered a broad range of countries and included the Thailand-United States FTA that was proposed but never concluded or signed. Three articles focused on IP provisions in the proposed Thailand-United States FTA and used prospective modelling to determine the welfare effect of TRIPS-plus provisions in the proposed text [25], the impact of proposed patent term extension, data exclusivity and patent linkage clauses on access to medicines [26] and the impact of proposed data exclusivity on the price and accessibility of medicines in Thailand [27]. These studies found that TRIPS-plus provisions in the proposed Thailand-United States FTA would have resulted in consumer welfare^6^ losses [25], an increase in drug expenditure and a delay in the market entry of generics [26, 27].

Two articles used retrospective quantitative analysis to measure the impact of data exclusivity clauses in the US-Jordan FTA on the availability of generics and the cost of medicines [28] and the additional cost to the private market due to delayed entry of generics [39]. The studies found that data exclusivity clauses delayed market entry of generics and increased costs for private consumers in Jordan [39] as well as preventing generic competition resulting in greater pharmaceutical expenditure by governments and consumers [28].

Two articles undertook prospective analyses to measure the welfare effects of the proposed IP clauses in the Korea-United States (KORUS) FTA [29] and the impact of KORUS on the timely availability of new medicines in Korea [30]. The findings suggest that patent term extension and patent linkage provisions in KORUS would result in total welfare losses, albeit smaller than previous studies have suggested [29]. Additionally, a retrospective analysis found that the KORUS FTA did not increase the availability of new medicines or reduce the time difference between drug approval in the USA and Korea [30].

Three prospective modelling studies focused on the impact of the two-year extension of biologics data exclusivity that was originally proposed by the USA for the Canada-US-Mexico Agreement (CUSMA)^7^ [24, 31, 32]. One study combined this analysis with the impact of Canada implementing patent term extensions (SPCs) as part of CETA [24]. All three studies reported that a two-year extension of biologics exclusivity in Canada would delay the market entry of generics and increase pharmaceutical costs [24, 31, 32]. The impact would be minor on products whose data exclusivity periods had expired [31].

One article retrospectively analysed the impact of the data exclusivity clause in the Central American Free Trade Agreement (CAFTA) on medicine prices in Guatemala [33]. It found that data exclusivity provisions resulted in reduced access to generic drugs already on the market and delayed the entry of new generics [33]. Another retrospective study examined the impact of all Chilean trade agreements on the volume and price of all biologics imported into Chile. This study found that the FTAs with more stringent IP provisions increased both the volume and the price of imported biologics [34].

A prospective analysis of the proposed Trans-Pacific Partnership Agreement (TPP)^8^ examined the potential impact of proposed IP provisions on access to anti-retroviral (ARV) medicines in Vietnam. It found that the proportion of people living with HIV, who receive ARV, would decrease if all IP provisions were implemented and funding remained constant [35].

The last article in this theme was a prospective modelling study that explored whether pharmaceutical spending increased in countries that signed FTAs with the USA between 1985 and 2016. In countries with US FTAs, it found that national drug spending had not increased as a share of overall health expenditure, the consumption of pharmaceuticals had not declined and there was no discernible shift to patent medicines from cheaper generics [36].

### Use of TRIPS flexibilities

Seventeen articles examined the impact of two TRIPS flexibilities on access to medicines in terms of availability, cost or price. We have divided these articles into those that examined compulsory licencing (*n* = 12), and those that examined parallel importation (*n* = 5). See Table 2 below.

**Table 2:** Studies of TRIPS flexibilities

Theme	Author(s), date and reference number	Study Type/Methodology	Key relevant findings
**Compulsory licencing**	**Thailand**		
	Mohara et al. 2012 [40]	Retrospective study to assess the impact on drug expenditure resulting from the introduction of the Government Use Licence policy in Thailand.	The use of generic drugs under the policy could save the government budget approximately $US370 million over 5 years. The compulsory licence for Efavirenz resulted in the biggest cost savings- $US116.45–118.84 million.
	Yamabhai et al. 2011 [41]	Prospective modelling (some data is actual impact- most is prospective) to measure the health and health-related economic impacts of seven government use compulsory licenses issued between 2006 and 2008.	The granting of the government use licenses resulted in an additional 84,158 patients estimated to have received access to the seven drugs over 5 years. Health gains from the use of the seven drugs compared to their best alternative accounted for 12,493 Quality-Adjusted Life Years (QALYs) gained, which translates into quantifiable incremental benefits to society of $US 132.4 million. The government use license on efavirenz was found to have the greatest benefit.
	Yamabhai and Tantivess 2009 [42]	Prospective study aims to assess impact of the government use licenses for seven drugs issued over the period 2006-2008 in Thailand. The assessment of the public health impact of the government use licenses is intended to determine or estimate the actual and expected increase in number of patients with access to the relevant drugs, and the public health benefits derived from such increased access, in terms of gains in patients’ health utility, measured in Quality-Adjusted Life Years (QALYs) gained or Disability-Adjusted Life Years (DALYs) averted. It also estimates economic impact.	The study estimated the increase in the number of patients with access to Efavirenz and Lopinavir/ritonavir over the five-year period to be 17,959 and 3421, respectively. The estimated increase in patients using clopidogrel was estimated to be 40,947. For the anti-cancer drugs, the estimates are as follows: 8916 patients for letrozole; 10,813 for docetaxel, 1846 for imatinib; and 256 for erlotinib. The results, in terms of QALYs gained (in order of drugs with the greatest health gains) were: 1. letrozole: a gain of 3656 QALYs; 2. EFV: 2694 QALYs gained; 3. clopidogrel: 2457 QALYs gained; 4. imatinib: a total of 2435 QALYs gained (1384 QALYs for Chronic Myeloid Leukemia (CML) patients; 1051 QALYs for Gastrointestinal Stromal Tumor (GIST) patients); and 5. docetaxel: 1251 QALYs gained. The use of the generic versions of the six original drugs by way of government use licenses would result in a reduction in national health expenditure, with estimated cost savings of approximately US$ 357.8 million for the 5-year timeframe. The impact was assessed in terms of the incremental benefits to health, which was estimated to be approximately $US 132.4 million for the 5-year study timeframe.
	**Brazil**		
	Scopel and Chaves 2016 [43]	Retrospective study to analyse the history of the price of lopinavir/ritonavir (LPV/r) in Brazil and in the international market including initiatives to challenge patent barriers between 2001 and 2012.	Between 2001 and 2003, there were efforts to use compulsory licensing as a threat. From 2005 to 2007, initiatives by different stakeholders were identified: declaration of public interest, pre-grant opposition and civil action. From 2006 to 2008, compulsory licensing initiatives in other countries resulted in a price reduction in Brazil. Between 2009 and 2012, there was a 30% reduction in the Brazilian purchasing price but can’t quantify price reduction to specific IP measures.
	Ramani and Urias 2018 [44]	Retrospective review to determine the possible inter-temporal impacts of catch-up in industrial capability on access to life saving drugs and vice versa in Brazil and what insights can be gained from the interrelationships between price negotiations of essential patented drugs, access to these and industry catch-up.	Industrial capability can provide bargaining strength in price negotiations and have a positive inter-temporal impact on both future industry catch-up and future access to essential medicines. Both the threat of and the grant of CLs result in price reductions.
	**Other countries**		
	Ortiz-Prado et al. 2019- Ecuador [45]	Retrospective study to identify and evaluate the possible effects of requesting compulsory licenses (denied, issued and withdrawn) on prices’ variability over the years after requesting them.	Ecuadorian experience shows that the use of Compulsory Licenses as a strategy for improving access to medicines requires more than political will. Offering better access to medicines requires adequate planning, intersectoral articulation, prioritization of goals, clear procedures and protocols and increased accountability. In the future, Ecuador should foster these capacities before initiating new CLs.
	Chatterjee et al. 2015- India [46]	Prospective study to assess the consumer welfare implications of changes in government policies related to patent protection and compulsory licensing in the Indian market for oral anti-diabetic medicines.	Under Scenario 4 (CL of all DPP-4 inhibitors), consumer welfare increases by around INR 47 million (USD 1 million). Scenario 5 – CL of sitagliptin and non-entry of other DPP-4 inhibitors – a negative response to CL by patent holders (deciding not to launch their products in India) can lead to a substantial loss in consumer welfare (consumer welfare falls by INR 141.5 million (USD 3 million) per year. Scenario 6 – CL of sitagliptin and vildagliptin, non-entry of saxagliptin – similar picture (consumer surplus falls by INR 8.3 million (USD 0.17 million) per year. Overall: Differential pricing and voluntary licensing improve consumer welfare. Issuance of CL also increases consumer surplus, but if it discourages firms from launching patented products and if local firms are unable to have their version of the patented drugs approved by the govt, there may be a decrease in consumer welfare. (Note: there is no direct evidence that CL drives off innovators from launching drugs in India – the paper simply simulates the effects on consumer welfare if this happens)
	**Global**		
	t’Hoen et al. 2018 [47]	A literature review to document the use of TRIPS flexibilities to access lower-priced generic medicines between 2001 and 2016.	Overall, 176 instances of the possible use of TRIPS flexibilities by 89 countries were identified: 100 (56.8%) involved compulsory licences or public non-commercial use licences and 40 (22.7%) involved the least-developed countries pharmaceutical transition measure. The remainder were: 1 case of parallel importation; 3 research exceptions; and 32 non-patent-related measures. Of the 176 instances, 152 (86.4%) were implemented. They covered products for treating 14 different diseases. However, 137 (77.8%) concerned medicines for HIV AIDS related diseases.
	Cherian 2016 [48]	Literature review to evaluate outcomes and policy approaches used by different countries for compulsory licenses under TRIPS Article 31, and identify shortfalls and best practices. Determined how Compulsory Licensing has used to enable generic entry and availability in some focus countries, and how use of the flexibility can set a precedent by informing the international regime on patents and the innovation framework.	Following the Doha ministerial declaration on public health in 2001, there has been more frequent use of compulsory licenses to procure HIV medications, and increasingly, non-essential medicines such as oncologic agents, anti-inflammatory agents, and prophylactic drugs for heart disease. Approaches taken by countries include an official Government-use policy to compulsory license drugs, use of CLs as a threat, an emergency use for pandemic preparedness, and anti-competitive tool to promote parallel trade. Each case has unique motivators and reveals context specific outcomes. Countries have successfully used CLs to increase access, provide costs savings and negotiate price reductions. After issuing a compulsory licence, a 73% price saving from purchasing generic Oseltamivir was seen in Taiwan and a 97% cost saving was seen in India from being able to purchase generic Sorafenib.
	Son and Lee 2017 [49]	A literature review to examine patterns and trends in attempts to issue compulsory licenses for pharmaceuticals, and to assess the related implications for access to medicines.	There have been 108 attempts to issue compulsory licensing for 40 pharmaceuticals in 27 countries since 1995. Most of the attempts were in Asian, Latin American, and African countries and mainly for HIV/ AIDS medicines. When the claimer was the government, the likelihood of approval and positive outcomes increased. Even the request for a compulsory licencing led to a price discount in 25% of cases.
	Beall and Kuhn 2012 [50]	Literature review to systematically analyse CL activity since the Doha declaration.	Twenty-four verified CLs were issued in 17 nations between 2001 and 2010. CL activity has diminished markedly since 2006. While upper middle income countries have high CL activity and strong incentives to use CLs compared to other countries, there is considerable countervailing pressures against CL use even in UMICs. The vast majority of CL episodes ended in some kind of price reduction via either CL, voluntary licence or discount.
	Beall and Attaran 2017 [51]	Retrospective study to determine whether developing countries, that have granted patent protection on essential ARVs, are procuring generic equivalents of those same medicines. Determine which legal flexibilities have been most relevant for facilitating access to medicines.	Voluntary licensing agreements between originator and generic companies resulted in more generic procurement than the other flexibilities (least developed country waiver, non-assert declarations and compulsory licences). Least developed country waivers also played an important role.
**Parallel importation**	**Multiple country EU**		
	Kanavos and Costa-Font 2005 [52]	Retrospective study to analyse the impact of parallel importation on stakeholders; the extent of competition in markets subject to PI, explain the overall determinants of PI and impact on prices, and determinants of price competition in PI importing countries.	There is an increase over time in parallel import market share in destination countries, but little price difference compared to domestic products. Key determinants of parallel trade penetration are price difference between destination and export country and pharmaceutical market size in importing country. Also important are the number of physicians/ 100 pop, and generic penetration. Parallel trade does not lead to lower prices in destination countries; most gains accrue to distribution chain, including pharmacies, some modest gains (< 2%) go to pharma funders (governments/insurers).
	Kyle et al. 2008 [53]	Retrospective study to examine the effect of parallel trade on patterns of price dispersion for prescription drugs in the European Union.	The distribution of prices among EU countries did not show a dramatic change concurrent with the adoption (especially after 1995) of parallel trade. About half of the price differentials in prescription drugs exceeded 50% in all EU and non-EU countries in each time period. Although price differentials decreased after 1995 for most countries, they decreased less in the EU than elsewhere.
	**Single country EU**		
	Duso et al. 2014- Germany [54]	Prospective economic modelling study to assess the welfare impact of parallel imports for all 700 anti-diabetic drugs sold in Germany between 2004 and 2010.	Parallel imports reduce the prices for patented drugs by 11% and do not have a significant effect on prices for generic drugs. This amounts to an increase in the demand-side surplus by €19 million per year (or €130 million in total) which is relatively small compared to the average annual market size of around €227 million based on ex-factory prices. The variable profits for the manufacturers of original drugs from the German market are reduced by 37% per year when parallel trade is allowed, yet only one third of this difference is appropriated by the importers.
	Mendez 2016- Denmark [55]	Economic modelling simulation of the effects of banning parallel imports. Aims to identify and understand the effects of parallel imports on consumers’ consumption choices, government expenditures for pharmaceuticals, and producers’ strategies	Data are for the statin market in Denmark. Banning parallel imports leads to (i) an increase in variable profits for original producers and a decrease for generic firms, (ii) an increase in governmental health care expenditures, and (iii) a decrease in consumers’ welfare.
	Granlund and Koksal-Ayhan 2014- Sweden [56]	Retrospective econometric study to determine the effect of competition from parallel imports on prices of locally sourced on-patent drugs and to determine whether the 2002 Swedish mandatory substitution reform increased this competition.	On average, facing competition from parallel imports caused a 15-17% fall in price. While the reform increased the effect of competition from parallel imports, it was only by 0.9%. The reform, however, did increase the effect of therapeutic competition by 1.6%.

#### Compulsory licencing

Three articles focused on Thailand’s issuance of compulsory licences (CLs) from 2006 to 2008 [40–42]. A retrospective costing study found the Thai Government decreased their pharmaceutical expenditure by granting seven CLs which allowed for a shift from patented to generic drugs [40]. Two prospective studies measured the impact of the same CLs on health and the economy in Thailand [41, 42]. These studies estimated that the compulsory licences allowed increased numbers of patients to access these seven drugs which resulted in significant quality adjusted life years (QALYS) gained and a reduction in national health expenditure [41, 42].

Articles that focused on compulsory licensing in Brazil (*n* = 2) included a case study that mapped initiatives to challenge patent barriers to the drug lopinavir/ritonavir [43]. It found that the threat of compulsory licensing for lopinavir/ritonavir and compulsory licensing in other countries resulted in significant price reductions in Brazil [43]. Another Brazilian case study used a literature review and qualitative methods to investigate, inter alia, ARV price negotiations and the threat of, and issuance of, a compulsory licence on the price of ARVs. Both resulted in price reductions [44].

Another case study focused on the Ecuadorian experience of CLs denied, issued and withdrawn [45]. It compared the prices before and after the request for compulsory licences and found that of the five that were granted, only one resulted in a price reduction. The authors concluded that compulsory licensing can be a useful tool in negotiating drug price reductions but requires careful planning, procedures, protocols and accountability [45].

The final single country compulsory licensing study was from India. This prospective econometric study examined the consumer welfare implications of Indian compulsory licensing policies on the market for oral anti-diabetic medicines [46]. It found that compulsory licencing increases consumer surplus^9^ but if it discourages firms from launching patented products in India, then it may lead to a decrease in consumer welfare [46].

Four articles employed literature reviews to document the use of TRIPS flexibilities to access lower-priced generic medicines [47], how compulsory licensing has been used to enable generic entry and availability [48], the patterns and trends in compulsory licencing [49] and the impact of the Doha Declaration on compulsory licensing [50]. Compulsory licencing was the most commonly implemented TRIPS flexibility [47] and was most commonly used for the treatment and management of HIV and AIDS [47, 49, 50]. Countries have successfully used CLs to increase access, provide costs savings and negotiate price reductions [48]. After issuing a compulsory licence, a 73% price saving from purchasing generic Oseltamivir was seen in Taiwan and a 97% cost saving was seen in India from being able to purchase generic Sorafenib [48]. Son and Lee found that even the request for a compulsory licencing led to a price discount in 25% of cases [49]. Beall and Kuhn found that the vast majority of CL events ended in some kind of price reduction via either CL, voluntary licence or discount [50]. Most attempts at compulsory licencing were in Asia, Latin America and Africa [49] by upper middle income countries (UMICs) [50] and were more likely to be successful if initiated at the government level [49]. The frequency of compulsory licencing has decreased significantly since 2006 [50]. The remaining study in this theme linked datasets to assess what TRIPS flexibilities developing countries that have granted patent product protection use to procure generic ARV medication [51]. Of these flexibilities, voluntary licensing resulted in the largest percentage of generic procurement followed by the LDC waiver [51].

#### Parallel importation

The five articles in this theme were all European-focused.^10^ Two included a multi-country EU focus [52, 53] and the remaining three included a focus on Germany [54], Denmark [55] and Sweden [56]. Both multi-country EU studies were retrospective econometric studies that assessed whether parallel trade reduces the cost of prescription drugs [53]. Both suggested that parallel trade did not result in significant reductions in the price of medicines within EU countries [52, 53]. However the authors stressed that this did not mean that parallel trade did not have the potential to significantly impact prescription drug prices [53]. Key determinants of parallel trade included price differentials between trading countries, pharmaceutical market size in the importing country, number of physicians and generic penetration [52]. The single country econometric modelling studies aimed to calculate the welfare impact of parallel imports of anti-diabetic drugs [54]; to investigate the impact of parallel trade in markets for pharmaceuticals [55]; and to determine the effect of parallel imports on prices of local patented drugs [56]. In contrast to the multi-country EU studies, findings suggest parallel imports reduce prices for patented drugs [54, 56] but do not affect the price of generic drugs [54]. In addition, without provisions for parallel importation there would be an increase in profits for originator firms, a decrease for generic firms, an increase in governmental health care expenditure and a decrease in consumer welfare [55].

### Patent expiry/generic entry/generic pathway

This theme of patent expiry, generic market entry and/or generic pathway was divided into studies that compared and contrasted multiple countries (*n* = 5) and those that focused on a single country (*n* = 18). See Table 3 below.

**Table 3:** Studies of patent expiry, generic entry and or generic pathway

Theme	Author(s), date and reference number	Study Type/Methodology	Key relevant findings
**Comparative country studies**	**EU Member States**		
	Elek et all 2017 [57]	Retrospective longitudinal study to determine whether the availability of generics improves medicine accessibility for patients and thus improves health outcomes. Focuses on clopidogrel.	Clopidogrel use increased significantly in lower income countries after generic entry but changed very little in higher income countries. Earlier generic entry has larger impact.
	European Commission 2009 [58]	Retrospective study to determine the reasons for observed delays in the entry of generic medicines to the market and the apparent decline in innovation as measured by the number of new medicines coming to the market.	Behaviour and practices of the originator industry contributed to delays in generic entry as well as to the difficulties in innovation, though there are other possible causal factors, such as regulation. It takes more than 7 months for generic entry to occur once originator medicines loose exclusivity. For the highest selling medicines, it took 4 months on average before market entry. Generic products, on average, enter the market with a price 25% lower that of originator medicines before the loss of exclusivity. Two years after entry, prices of generic medicines were on average 40% below the former originator price. The prices of originator products also appear to drop following generic entry. The market share of generic companies was about 30% at the end of the first year and 45% after 2 years. In other words, any delay in generic entry will have a significant cost / revenue impact. Savings due to generic entry could have been 20% higher if entry had taken place immediately following loss of exclusivity. The aggregate expenditure amounting to about €50 billion for the period after loss of exclusivity would have been about €15 billion higher without generic entry. However, additional savings of some €3 billion could have been attained, had entry taken place immediately.
	**Other countries**		
	Morton 2018- [59]- EU and Australia	Prospective econometric modelling to analyse European markets, which have had biosimilar competition since 2006 and how market features and public policies predict biosimilar entry, price, and penetration.	Focuses on markets where there are tenders for the supply of medicines. Prevailing market prices fall over time at an average rate of about 3.5 percentage points per year following biosimilar entry, and this decline is even steeper in Epoetin and Filgrastim markets. Price declines are steeper when there are more biosimilar entrants and when the size of the tender population is larger. Quotas for biosimilars seem to raise their prices in some cases, as one might expect if the quota regulation reduces supply.
	Berndt and DuBois 2016 – EU and North America [60]	Retrospective study to determine the effect of patent expiry on daily cost of pharmaceutical treatments, trends over time, and factors affecting variations in price indexes per day of treatment across countries, therapeutic classes, and time.	Prices have fallen over time, as generics enter average prices fall more in pharmacy-driven than in physician-driven markets. Regulatory and pricing policies – particularly those at the level of therapeutic class – have expected effects on average prices. Greatest falls in 4 of the 10 therapeutic classes: ace inhibitors (−19%), anti-ulcerants (−13%), calcium channel blockers (−12%), and lipid regulators (−11%). There is clear evidence on the downward evolution of prices for therapeutic classes experiencing patent expiration over the 2004–2010 time period.
	Liu and Galarraga 2019 – Southern Africa [61]	Retrospective study to estimate the relationship between ARV drug prices and national drug policies with a focus on countries in the Southern African Development Community.	The most consistent predictor of ARV drug price is a drug’s generic/patent status. The generic versions of 8 out of 10 ARV drugs were priced lower than branded versions. There was no consistent association between ARV prices and transaction volume, national drug policies or Presidents Emergency Plan for AIDS Relief/ Clinton Health Access Initiative involvement.
**Single country studies**	**US studies**		
	Branstetter 2016- USA [62]	Retrospective econometric modelling to determine the welfare effects of accelerated generic entry via Paragraph IV challenges.	Consumers gain $42 billion whereas producers lose $US32.5 billion from accelerated generic entry as a result of Para-IV challenges. Overall consumption does not increase after entry—generic sales displace branded sales, shifting surplus downstream from producers to consumers, insurance companies, and retailers.
	Grabowski 2016- USA [63]	Retrospective econometric modelling to determine the proportion of new drug introductions subject to patent challenges and the time to patent challenge in the wake of the 1998-2003 legal and regulatory changes to the Hatch-Waxman Act.	For small molecule drugs only: the proportion of patents challenged increased from 34% in 1995 to 79% in 2003 (69% for core patents). Large sales small molecules: For active ingredient patents, in 45% of challenges generics win. For method of use patents, generics win 79%, and for formulation patents generics win 97%. A large proportion of challenges are settled for all patent types. Biologics: 8% of patents challenged compared to 34-69% of small molecule drug patents, depending on year.
	Berndt and Aitken 2011- USA [64]	Retrospective study of the effect of the Waxman-Hatch legislation on generic market share, the generic price index, the average price per prescription and the daily cost of treatment.	Generic share grew from 18.6% in 1984 to 74.5% in 2009. Average price per prescription fell by 21.3% from 2006 to 09. Weighted mean reduction in pharmaceutical daily treatment cost across nine therapeutic areas was 35.1% at 24 months post-generic entry. Generic share has increased, and prices have declined dramatically since the Waxman- Hatch legislation.
	Bokhari and Fournier 2013 – USA [65]	Retrospective econometric modelling of the potential welfare gains due to the introduction of generic psychostimulant (ADHD) drugs as well as of me-too’s and the welfare loss due to the delayed entry of generics in this market.	Both Concerta and Adderall extended release (XR) created new niche markets for their respective molecules by introducing effective new delivery mechanisms. Consumers placed a large value on these improvements, on average approximately $US137 and $US123 per child per year respectively, and consequently these two me-too drugs achieved 24 and 26% of the ADHD drug market. The introduction of generic Adderall in the MAS-IR segment extended the market and was very valuable to consumers (about $US65 per child per year). Shire holds two key patents on Adderall XR that technically prevent entry in the MAS-ER segment until 2018 and an exclusivity period until April 2005.
	Castanheira 2017- USA [66]	Retrospective econometric analysis to determine the effect of generic entry on price and demand (volume) of medicines.	After generic entry, the price of the molecule drops, on average, by about 45% after 3 years. The market share of the molecule drops by around 25-30%. That is, the cumulated sale volume of the original brand plus its generic competitors drops, even though these markets are typically growing.
	Frank 1997- USA [67]	Retrospective econometric modelling to estimate price responses to generic entry in the market for brand-name and generic drugs.	Brand-name prices increase after generic entry and are accompanied by large decreases in the price of generic drugs.
	Huckfeldt and Knittel 2011- USA [68]	Retrospective econometric modelling to estimate the effects of generic entry on prices and utilization of blockbuster drugs in 4 therapeutic areas. Also examines how generic entry effects differ by sources of health insurance.	Generic prices fall relative to branded prices, and branded company market share falls rapidly after the entry of generics. Demand for the branded molecule begins to fall prior to generic entry and coincides with increased use of branded reformulations – a shift to a slightly different product. These patterns exist across individuals with different sources of health insurance. Case study evidence implies that marketing to physicians and patients drives these patterns of use.
	Shih et al. 2007- USA [69]	Retrospective econometric modelling to explore the impact of generic drug entry on the cost-effectiveness of selective serotonin reuptake inhibitors.	There is a positive benefit from generic entry in all 4 selective serotonin reuptake inhibitors compared to paroxetine pre-generic entry (ie availability of alternatives had a positive price effect). After generic entry, price effectiveness compared to paroxetine is variable. However, the entry of a new drug - Escitalopram - had a stronger effect on market shares than the entry of generic versions of paroxetine.
	Hemphill 2012- USA [70]	Retrospective econometric modelling to determine whether generic patent challenges are on the rise and whether they reduce the effective market life of new medicines. Also looks at whether patent challenges disproportionately target high-sales drugs thereby reducing market life for these “blockbusters”.	The average nominal patent term is 16 years for drugs with first generic entry between 2001 and 2010. By comparison, average effective market life for these drugs is 12 years, not much different than in the previous decade, and greater than in the decade before Hatch–Waxman. Patent challenges are the key driver of the gap between nominal patent term and effective market life. Challenges are more prominent for large sales drugs and there is an increase in early challenges.
	Hemphill 2011- USA [71]	Retrospective econometric modelling of the Hatch-Waxman Act “Paragraph IV” challenges as a means to secure early market entry.	Over time, patenting has increased, measured by the number of patents per drug and the length of the nominal patent term. Meanwhile, the proportion of drugs subjected to patent challenges has increased. Drugs are also challenged sooner, relative to brand-name approval. Brand-name sales have a positive effect on the likelihood of generic challenge. The likelihood of challenge also varies with the nature of the patent portfolio. A drug with weaker patents faces a significantly higher likelihood of challenge, conditional on sales and other drug characteristics. That is not because the drug’s patent protection is weaker overall; additional patents, even weak ones, generally strengthen a brand-name firm’s ability to exclude. Rather, a weak patent, particularly if it expires later than the basic patents, disproportionately attracts a challenge to the pertinent drug. Overall, results suggest these challenges serve a useful purpose by promoting scrutiny of weaker and late-expiring patents.
	**Canada**		
	Lexchin 2004– Ontario, Canada [72]	Retrospective study to examine the effects of the entry of generic competitors on the price of brand-name products in the province of Ontario.	Price changes for 81 different products in 144 separate presentations were analysed. There was no statistically significant change in brand-name prices when generic competition started. The movement of brand-name prices was not influenced by whether the generic was made by the company producing the brand-name product or price freezes imposed by the Ontario government. When generics first became available having four or more generics was associated with a rise in the price of the brand-name drugs compared to having one, two or three generic competitor(s).
	Jones et al. 2001- Canada [73]	Retrospective analysis of the impact of 1987 and 1993 changes in Canadian legislation determining length of patent monopoly.	Despite evidence of significant first mover advantages which resulted in higher brand prices, competition from generics succeeded in reducing overall market prices prior to 1987; but after 1987, the efficacy of generic competition was reduced and both brand and market prices increased. First, prior to the 1987 amendments to the Patent Act, generic competition succeeded in reducing the overall (brand and generic) market price of ethical drugs in British Columbia. This was due entirely to competition among generic drug producers, because branded drug prices continued to increase demonstrating robust first mover advantages. Second, after 1987, when the period of market exclusivity was extended for branded drugs, the moderating effect of generic competition was reduced.
	**Other countries**		
	Gleeson et al. 2019- Australia [74]	Retrospective to estimate the potential savings to the Pharmaceutical Benefits Scheme (PBS) and the Repatriation Pharmaceutical Benefits Scheme (RPBS) in 2015–16 if biosimilar versions of selected biologic medicines had been available and listed on the PBS.	Australian Government expenditure on biologics on the PBS and RPBS was estimated at A$2.29 billion dollars in 2015–16. If biosimilar versions of these medicines had been listed on the PBS in 2015–16, at least A$367 million dollars would have been saved in PBS and RPBS subsidies. Modelling based on price decreases following listing of biosimilars on the PBS suggests that annual PBS outlays on biologics could be reduced by as much as 24% through the timely introduction of biosimilars.
	Hill et al. 2017- UK [75]	Prospective modelling to estimate lowest possible treatment costs for four novel cancer drugs, hypothesising that generic manufacturing could significantly reduce treatment costs.	Generic production could allow the UK price of dasatinib to decrease by 99.6%, and the UK price of gefitinib to decrease by 99.5%. Importation of Indian generics would represent a UK price decrease of 74% for bortezomib and 71% for everolimus.
	Kaier 2012- Germany [76]	Retrospective analysis to determine whether demand in Germany for specific antimicrobial agents is driven by prices that drop considerably when generic substitutes become available.	Patent expiration is followed by substantial decreases in the price of antibiotics. In the outpatient sector, all antibiotics included in the analysis showed significant negative own-price elasticities of demand. However, in the hospital settings, significant own-price elasticities were only determined for some antibiotics, although price decreases were stronger than in the outpatient sector. Price dependence of demand for antimicrobials is present both in the ambulatory and the hospital setting.
	Boersma et al. 2005- The Netherlands [77]	Retrospective analysis to investigate the influence of patent expiry on the use and cost of three selected drugs in the Netherlands, for which patents expired between 1996 and 2001.	For two of the drugs, there was a significantly steeper decline in the cost per defined daily dose after patent expiry (for the third, there was too little data to estimate the trend before patent expiry). For the three drugs, the cost per DDD fell by 61, 51 and 69% after patent expiry. After patent expiry, generics accounted for 75% of the drugs dispensed. Patent expiry causes significant price competition that leads to an overall decrease in costs.
	Manova et al. 2010- Bulgaria [78]	Retrospective analysis to determine the effects of the arrival of generic and/or therapeutic competitors on the market, in terms of the impact on the market share and prices.	Generic competition, in general, changes the market. These changes decrease the price of the medicines. The introduction of generics changes the market share of the originator brand medicines and contributes to the changes in the share of the therapeutic groups. Differences in prices within the same drug classes but no significant change across the years.
	Asif et al. 2019- Pakistan [79]	Retrospective study to empirically evaluate the trends of induction of novel drugs (i.e. new molecular entities and generic drugs) post TRIPS in Pakistan.	The results indicate a marked and consistent decline in the entry of novel drugs after TRIPS implementation, indicating that the access to novel medicines issue is likely amplified rather than improved by giving patent rights for the pharmaceutical products.

#### Comparative country studies

Two statistical modelling studies focused on EU Member States [57, 58] and one on EU Member States and Australia [59]. These studies explored whether generic availability improves affordability for patients and improves health outcomes [57] and determined the cost savings from the market entry of biosimilars [59]. The findings indicated that drug use is strongly influenced by price. This was more notable in lower income than higher income EU countries. Early generic entry was associated with a larger price impact [57]. Similarly, upon market entry, generic drugs were significantly more affordable than originator drugs and this difference grew over time. Generic market share also increased over time despite the originator drug price falling following generic entry [58]. The behaviour of originator firms delayed generic entry and contributed to a decrease in innovation and led to reduced health savings [58]. Morton et al. estimated that biosimilar entry resulted in savings of around $1.5 billion in 2006 in the EU and that average market prices fell at about 3.5 percentage points per year following biosimilar entry [59].

Another multi country retrospective econometric study included selected OECD countries in Europe and North America. It aimed to quantify the impact of patent expiry on the daily cost of pharmaceuticals [60]. Like the EU studies, it found that average medicine prices fall upon generic entry. The last study in this theme focused on the countries of the Southern African Development Community. This retrospective statistical study analysed global ARV prices to examine the relationship between national drug policies and ARV prices [61]. The most consistent predictor of ARV drug prices was generic status. In the vast majority of cases generic ARVs were priced lower than originator drugs [61].

#### Single country studies

Ten out of the 18 studies in this theme are from the USA. Four of these 11 studies involve analyses of aspects of the Hatch-Waxman Act 1984 including Paragraph IV [14].^11^ Two studies use statistical modelling to assess the welfare effects of accelerated generic entry via Paragraph IV challenges by generic companies [62], and the impact of regulatory changes to Paragraph IV patent challenges by generics on the effective market life of branded drugs [63]. A retrospective study analysed the effect of the Hatch-Waxman Act on generic market share, the generic price index, the average price per prescription and the daily cost of treatment [64]. Findings suggest that consumption does not increase after generic entry, however, generic sales displace branded drug sales. Financially, consumers gain, and producers lose from accelerated generic entry. Generic and branded drug prices decreased on average after generic entry [62]. The generic market share grew dramatically after the advent of the Hatch-Waxman Act. The average price of prescriptions fell as did daily treatment costs across various therapeutic areas [64]. Regulatory changes to the Hatch-Waxman Act that occurred in 1998-2003 led to increased patent challenges by generic companies for top selling drugs [63].^12^ This increase in challenges led to nominally reduced market exclusivity periods for branded drugs.^13^

Five studies used econometric modelling with US data to measure: the welfare impact of generic entry and the welfare loss of delayed generic entry [65], the effect of generic entry on price and demand of medicines [66], the effect of generic entry on market prices of originator and generic drugs [67], the effects of generic entry on prices and uptake of specific blockbuster^14^ drugs and how this entry effect differs under various health insurance schemes [68] and the impact of generic drug entry on the cost-effectiveness of a class of anti-depressant drugs [69]. These four studies found that after generic entry both the price and volume of sales drops (both originator and generic versions) due to reduced promotion by the originator company [66]. In contrast, Frank et al. found that originator drug prices increase after generic entry and generic prices continue to fall after more generic competitors enter the market [67]. Generic entry causes the market share of originator drugs to fall; however, this coincides with originator companies launching new branded reformulations which are promoted just prior to generic entry [68]. The entry of a generic paroxetine (an SSRI anti-depressant) lowered the price of the originator and reduced the cost-effectiveness of other anti-depressants in that class relative to paroxetine [69]. Bokhari et al. found that generic entry resulted in large welfare gains due to drug price reductions [65].

The final two US-based studies are by the same authors and use statistical modelling to determine whether patent challenges decreased the effective market life of branded drugs and whether challenges disproportionately target blockbuster drugs [70] and examined the relationship between patent portfolio building and the likelihood of a Paragraph IV challenge [71]. Findings indicate that the average nominal patent term is 16 years and the effective market life of these drugs is 12 years. This is not markedly different to patent term and market life durations prior to the introduction of the Hatch-Waxman Act [70]. Patent challenges by generic companies are the main reason for the gap between patent duration and effective market life and challenges are more common for blockbuster drugs [70]. Despite the increasing prevalence of weak secondary patents, the number, nature and duration of listed patents have no clear impact on the duration of effective market life [60]. Hemphill and Sampat also found that while patenting has increased over time, so too has the fraction of drugs subject to patent challenges and the speed with which they are challenged [71]. A drug with weaker patents and higher drug sales is more likely to face generic challenge. Weaker patents are less likely to be related to socially valuable research and development and challenges to such patents might be an important means of ensuring the patent system is socially valuable [71].

The non-US studies in this theme are a mix of Australian, European and Canadian studies and one from Pakistan. Two retrospective studies from Canada examined the effect of generic entry on the price of branded medicines in the province of Ontario [72] and the impact of the 1987 and 1993 legislative changes regarding the price of drugs [73]. In Ontario, generic entry did not change the price of brand name drugs. However, the presence of at least four generic competitors was associated with a rise in brand name drug prices [72]. In British Columbia, prior to the 1987 legislative amendments, overall market prices reduced upon generic entry. After the amendments, generic competition was reduced and both brand and market prices increased [73].^15^

A retrospective study from Australia estimated the savings to the Pharmaceutical Benefits Scheme (PBS) if biosimilar versions of certain biologic drugs were listed and available on the PBS. It found that listing biosimilars could lead to considerable savings and PBS outlays could be reduced by up to 24% [74].

A UK prospective modelling study quantified the lowest possible treatment costs of four novel drugs to treat cancer and found that local generic production could reduce the price of treatment by over 99% and importing a generic from India could also significantly reduce the price of treatment [75]. A retrospective study in Germany determined whether demand for antimicrobial agents was driven by price drops following generic entry. It found that prices dropped significantly following generic entry in both hospital and outpatient settings and this impacted demand [76]. Another retrospective study from the Netherlands examined the use and cost of select drugs following patent expiration. They found large reductions in the cost of defined daily doses of the select drugs following patent expiry, with generic drugs accounting for a majority share of the market [77]. There was a similar finding in a Bulgarian retrospective study that analysed the impact of generic entry on drug prices and market share [78]. Generic competition changed the market and decreased the price of medicines [78]. The last study in this theme is a retrospective analysis from Pakistan of trends in the introduction of new molecular entities (NMEs) and first entry generics following the introduction of product patents. In the years following Pakistan’s signing of the TRIPS agreement, there has been a marked and consistent decline in the number of NMEs and first entry generics registered in Pakistan. Conversely, there were increasing numbers of new branding of old generics for the same time period [79].

### Patent policies

This theme includes studies that analyse the impact of specific patent policy provisions on access to medicines in terms of coverage, availability, cost or price. We have divided this theme into articles that compare and contrast multiple country settings (*n* = 9) and those that are focused on a single country context (*n* = 9). See Table 4 below.

**Table 4:** Studies of patent policies

Theme	Author(s), date and reference number	Study Type/Methodology	Key relevant findings
**Comparative country studies**	Cockburn et al. 2014 [81]	Retrospective econometric study to determine how patent and price control policies, as well as economic and demographic factors, affect the speed and scope of diffusion of new pharmaceutical products across countries.	Price regulation delays launch, while longer and more extensive patent rights accelerate it. Longer, and stronger, patent protection powerfully accelerates diffusion of new drugs. Controlling for economic and demographic factors, moving from a regime of no product patents to a long product patent term reduces launch lags by about 55%. Process patents also promote faster launch, but the impact is not as large as for product patents. Importantly, the impact of policy regimes holds equally for low- and middle-income countries as for high-income countries.
	Lanjouw 2005 [82]	Retrospective econometric study to determine how legal and regulatory policies affect whether new drugs are marketed in a country, and how quickly.	Less than one-half of the new pharmaceutical molecules that are marketed worldwide are sold in any given country, and those that are sold are often available to consumers in one country only 6 or 7 years after those in another. Both price regulation and IP rights influence these outcomes. A stronger patent regime is associated with more of the drug launch in a country being done by the originator firm. Overall, two-thirds of all drug launches and three-quarters of blockbuster launches are done by the originator firm.
	Watal and Dai 2019 [83]	Retrospective econometric study to investigate how the introduction of product patents in pharmaceuticals affects the likelihood of pharmaceutical firms to launch new and innovative medicines in those markets. Also looks at how much patent owners or generic pharmaceutical firms adjust their prices to local income levels.	The introduction of product patent for pharmaceuticals has a positive effect on launch likelihood, especially for innovative pharmaceuticals. However, this effect is quite limited in low-income markets. Also, innovative pharmaceuticals are launched sooner than non-innovative ones, irrespective of the patent regime in the local market. There is evidence of differential pricing for both originator and generic products. Overall, originators differentiate by about 11% and generics by about 26% between markets with different income levels. Differential pricing is larger for pharmaceuticals to treat infectious diseases, particularly for HIV/AIDS medicines, than for non-communicable diseases. However, pharmaceutical prices are far from fully adjusted to local income levels in either case. However, competition, especially that within a particular medicine market, can effectively drive down prices in both originator and generic markets.
	Kyle and Qian 2014 [84]	Retrospective econometric study to examine the consequences of stronger pharmaceutical patent protection on the speed of drug launch, price, and quantity in 59 countries from 2000 to 2011.	Patents are generally associated with faster launch, higher prices, and higher sales. The importance of patents varies across country / income groups.
	Borrell and Watal 2003 [85]	Retrospective econometric study to measure the impact of patents on unsubsidized sales of new ARV drugs in a sample of low and middle-income countries in the late 1990s.	On average patents increase availability of new drugs (from 28 to 33%), but patents reduce sales by 59% once the drug is available in the marketplace. The net effect of these two counterbalancing effects is that patents reduce sales by 34%. Switching all drugs from a patent regime to no-patent regime would increase the percentage of people living with HIV that are treated using new drugs by 34%. However, such an increase only shifts market coverage up from 0.88 to 1.18%.
	Hellerstein 2012 [86]	Retrospective econometric study to quantify the effects of drug monopolies and low per-capita income on pharmaceutical prices in developing countries using the example of ARVs used to treat HIV.	This paper compares markups on ARVs in countries with monopolistic drug markets to those with more widespread availability of generics. The data suggest consumers in richer countries pay more for drugs. Consumers also pay more when there is a monopoly supplier. Prices are around 20% of average income in competitive supply countries but over 90% in monopoly supply countries, making them less affordable.
	Jung and Kwon 2015 [87]	Retrospective econometric study to examine the effect of stronger IP rights on public health, especially on medicine use in low- and middle-income countries.	Higher level of IP rights is associated with low access to prescribed medicines. This adverse relationship between IP and access to medicines is significant even after controlling for country income level and individuals’ socioeconomic status and demographic characteristics. Adding other variables, which reflect the characteristics of each country’s healthcare system, did not change the significant effect of IP rights on access to medicines, although the magnitude of the effect slightly decreased.
	Rozek and Berkowitz 2005 [88]	Retrospective econometric study to determine how prices of branded and unbranded pharmaceuticals are affected by increasing IP rights.	Prices of existing branded pharmaceutical products do not change as a result of increasing IP protection. Price data for the 14 common drugs in all four countries that increased IP protection do not exhibit any pattern of price change after the IP policy change. Countries with IP protection do not systematically have higher prices than countries without. Prices across countries are largely dependent on launch dates.
	Djolov 2005 [89]	Retrospective econometric study to demonstrate the benefits of competition as supported by pricing freedom and strong property rights protection in pharmaceutical markets across the world.	On average, 98% of the population of countries with ‘strong’ pharmaceutical patent protection have access to essential drugs, with the corresponding figure for countries with weak patent protection being 76%. If countries with weak patent protection shift their protection to the levels of the countries with strong patent protection, 743 million additional people worldwide may be gain access to essential drugs. On average, countries with strong patent protection allocate 0.55% of their GDP to pharmaceuticals, relative to 0.70% for countries with weak patent protection.
**Single country studies**	**India**		
	Dutta 2011- India [90]	Prospective modelling to determine the changes in consumer surplus, profits, market prices, and market quantities that would result from pharmaceutical product patents and price deregulation for 43 drugs for which foreign firms were present in India.	For the 43 drugs considered, the total loss in consumer welfare from product patents and price deregulation is estimated to be $US 378.5 million, which amounts to an average loss of $9 million per drug and an overall decrease in consumer surplus of 48% from the free entry scenario. Product patents and price deregulation are together estimated to result in a total loss of almost 8.5 million patients, representing a decrease of over 50% from the free entry scenario. In contrast, the average annual gain to the foreign patent holder from this policy simulation exercise is estimated to be $US1.4 million per drug.
	Chaudhuri et l 2006- India [91]	Prospective modelling to determine the welfare effects (ie net impact on society) of pharmaceutical product patents.	The cost to consumers of product patents (US$255 m) is multiples more than the benefit to foreign patent-owning producers (US$53 m without price regulation and US$20 m with price regulation). It would be far cheaper to subsidise pharmaceutical company profits directly than to allow pharmaceutical product patents.
	Duggan et al. 2016- India [92]	Prospective modelling to determine the effects of the 2005 implementation of product patents in India on pharmaceutical prices, quantities sold, and market structure.	A molecule receiving a patent experienced an average price increase of 3–6%, with larger increases for more recently developed molecules and for those produced by just one firm. Results show little impact on quantities sold or on the number of pharmaceutical firms operating in the market.
	Watal 2000– India [93]	Prospective econometric modelling to estimate how much pharmaceutical prices would increase and welfare decrease following the introduction of product patents in India.	Overall maximum weighted price increase for the entire patentable pharmaceutical segment would be a mean of 26% with linear demand, and 242% with constant elasticity of demand. Additional welfare loses in moving from the current largely oligopolistic markets to patent monopoly would be $US50 million with linear demand and $US141 million with constant-elasticity-type demand function. These amount to about 3 and 8% of the total pharmaceutical market respectively. Consumers would lose, in terms of consumers’ surplus, anything between 11 and 67 million at the maximum, depending on the type of demand function assumed. Price increases are the highest for the product where price elasticity is the least.
	Berndt and Cockburn 2014- India [94]	Retrospective econometric study to examines the impact of patent policy in India on the availability of new drugs (by measuring the launch lag in comparison with other countries).	There is an estimated launch lag of 4.5-5 years in India, compared with 1 year in German and 2 months in the US. Significant numbers of drugs took much longer than mean/medium time to launch. Ten years after the first worldwide approval, almost a quarter of the sample drugs were not available in India.
	**Other countries**		
	Challu 1995- Italy [95]	Comparative statistical analysis of the impact of pharmaceutical product patents on i) pharmaceutical prices; ii) pharmaceutical inventions; iii) balance of pharmaceutical trade.	Compares prices for drugs available in Italy before and after pharmaceutical product patents were introduced. For drugs marketed before the introduction of product patents, average Italian prices were 54% lower than current prices in the USA. For drugs introduced in Italy after product patents, prices are 67% higher than in the USA.
	Yamabhai and Smith 2015 – Thailand [96]	Retrospective econometric study to assess the relative impact of patent status as a component of pharmaceutical prices, while controlling other market and medicine characteristics.	Data are for retail prices of oncology medicines in Thailand. Patented status is associated with a price of approximately 144-206% more than that of an equivalent generic. Larger sales volumes, a more competitive market and a longer product life are also associated with prices around 3-30% higher.
	Grootendorst et al. 2018- Canada [97]	Retrospective econometric study to investigate whether Canada’s several IP changes had an effect on the market exclusivity duration of brand products on the Ontario Drug Benefit formulary.	There were 595 drugs launched between 1974 and 2012 that were available for analysis. Exclusivity gradually declined from the late 1970s to 1990. Drugs approved in 2004 received 7.6 years of exclusivity, and drugs approved in 2005 received 5 years of exclusivity. Over the time period analysed, market exclusivity time of brand drugs experienced marked changes, but no systematic effects of Canada’s stronger pharmaceutical IP laws on the market exclusivity were detected.
	Arcidiacono et al. 2013- USA [80]	Retrospective econometric study to estimate consumer welfare effects of generics and “me-too” drugs, taking into account the intervening role of insurance.	Generics and “me-too” drugs each increased consumer welfare more than $US100 million in 2010, holding insurance premiums constant. Insurance payments in 2010 fell by nearly $US1 billion due to generics and rose by over $US7 billion due to me-too antiulcer drugs.

#### Comparative country studies

Articles in this theme include multi-country studies that span between nine and 76 countries. Four econometric retrospective studies examined the impact of patent policies on various aspects of drug launches [81–84]. A study of 68 countries assessed how regulatory polices affect whether new drugs are launched in a country and how quickly [82]. Similarly, three studies investigated how patent regimes affect the timing of drug launches in 76 countries [81], the speed of drug launch, price and quantity of drugs sold [84] and how the introduction of product patents affect the likelihood that pharmaceutical firms launch new and innovative medicines [83]. This study also measures how much patent owners or generic firms modify their prices to local income levels in 70 countries [83]. Findings suggest that longer patent protection facilitates market entry of new drugs in high income countries, though the evidence is mixed as to whether longer patent periods improve access to new medicines in LMICs [82]. Similarly, Watal and Dai found that pharmaceutical product patents facilitated the likelihood of drug launch, though in low-income markets this effect is limited. Innovative medicines are launched sooner than non-innovative ones and differential pricing does not appear to be adjusted to local income levels [83]. These findings were reinforced by Cockburn et al. who found that longer and more extensive patent provisions facilitate launches of new drugs; however, in contrast to the other studies the findings were equally applicable to low- and middle-income countries as high-income countries [81]. Similarly, Kyle and Qian found patents to be associated with earlier launch of new products, higher drug prices and higher sales volumes. However, they also found that the increase in price associated with patents was smaller in lower income countries and suggested that policies to countervail price increases were therefore effective [84].

Two retrospective modelling studies aimed to assess the impact of patent provisions on access to ARV medicines in low income countries [85, 86].^16^ The findings suggest richer countries may pay a little more for ARVs, but a lot more if an originator brand is supplying them [86]. These findings are supported by Borrell and Watal who found that pharmaceutical product patents constrain the sale of new drugs in developing countries and that switching all ARVs to generics would increase the coverage of treatment with new drugs of people living with HIV considerably [85].

Two retrospective studies analyse the impact of stronger patent laws on access to medicines and catastrophic health expenditure for medicines in 35 LMICs [87] and the impact of patent provisions on the price of pharmaceutical products in nine middle and LMICs [88]. Although these studies shared similar aims, their results differed. Jung and Kwon found that stronger IP laws (as evidenced by a higher Ginarte Park index score^17^) significantly decreased access to medicines even after controlling for country income levels and individual wealth [87]. Rozek and Berkowitz report findings to the contrary. Their analysis finds that existing prices of branded pharmaceutical products did not exhibit any price change after the introduction of ‘stronger’ IP laws and that countries with stronger IP regimes did not have higher pharmaceutical prices than those without IP regimes [88].

The last article under this theme was a modelling study that aimed to link the strength of IP regimes with access to essential drugs using data from 55 countries [89]. The study found that 98% of the population of countries considered to have strong patent regimes have access to essential drugs in contrast to 76% of people in countries with weak patent regimes. Djolov concluded that many millions more people would gain access to essential drugs if countries with weak patent regimes reformed them to be at the level of countries with strong IP [89].

Articles using ‘strong IP regimes’ as the key independent variable rarely define the precise meaning of this term, limiting the usefulness of their findings.

#### Single country studies

The majority of the single country studies in this theme are from India. Many focus on the introduction of pharmaceutical product patents in India in 2005. Two prospective econometric studies investigated the welfare effects of pharmaceutical product patents and price deregulation [90, 91]. Both studies found that for the class of drug they assessed, product patents and price deregulation would result in large losses in consumer welfare and welfare losses for the Indian economy [90, 91]. Only a small fraction of these losses would be due to increased foreign pharmaceutical company profits [90, 91]. Another modelling study investigated the impact of product patents on drug price and quantities sold [92] and a prospective study measured the increase in pharmaceutical prices and decrease in consumer welfare following the introduction of product patents in India [93]. Both studies found that product patents cause drug prices to rise [92, 93]; however, Duggan et al. found that these increases were larger for recently developed medicines and for those with few therapeutic substitutes [92]. Watal et al. also reported significant welfare losses from the introduction of product patents [93]. The last article from India in this theme was a retrospective quantitative study that investigated the impact of patent policy in India on the availability of new drugs by measuring delays in drug launch [94]. New drugs in India had an estimated launch lag of 4.5-5 years, a much greater period than Germany or the USA. This lag time is greatly reduced for blockbuster drugs [94].

A retrospective = study from Thailand and an comparative study from Italy aimed to assess the impact of patent status on pharmaceutical prices [95, 96] and pharmaceutical inventions [95]. Both studies found that patented medicines were associated with considerably higher prices than equivalent generics [95, 96].

A study from Canada examined whether IP changes over 3 decades affected the market exclusivity time of brand products on the Ontario Drug Benefit formulary [97]. Over the time period analysed, the study did not detect any systematic effects of Canada’s stronger pharmaceutical IP laws on market exclusivity [97].

The last study in this theme is a retrospective modelling study from the USA. It investigated the welfare effects of generics and me-too^18^ drugs [80]. Both generics and me-too drugs increased consumer welfare, however, insurance payments decreased for generics but increased for me-too drugs. Removing generics from the market would decrease consumer welfare and increase insurance payments [80].

### TRIPS plus rules

This theme includes studies that analyse the impact of particular TRIPS-plus rules on access to medicines in terms of availability, cost or price. We have divided this theme into articles that focus on the impact of data exclusivity (*n* = 3), patent term extensions (*n* = 3) and those that focus on secondary patents (*n* = 9). See Table 5 below.

**Table 5:** Studies of TRIPS-plus rules

Theme	Author(s), date and reference number	Study Type/Methodology	Key relevant findings
**Data exclusivity**	**USA**		
	Kesselheim and Solomon 2010 [98]	Retrospective study to explore increases in the cost of old drug Colchicine arising from market exclusivity.	After the FDA approved Colcrys, the manufacturer brought a lawsuit seeking to remove any other versions of colchicine from the market and raised the price by a factor of more than 50, from $US0.09 per pill to $US4.85 per pill. Use of the new brand-name colchicine could add as much as $US50 million per year to insurance programs’ budgets. The implications of market exclusivity for public health can be substantial.
	Nelson et al. 2011 [99]	Retrospective econometric modelling to estimate the cost impact of the 6-month exclusivity extension policy on the Utah Medicaid drug programme. Also projects the cost impact of this policy on Medicaid programmes in the US during the 18 months following patent expiration.	The 6-month extension policy was estimated to cost Utah’s Medicaid $US2.2 million for three drug classes over the 18 months following the original patent expiration date (year 2007 values). Projected to the US Medicaid population, this cost was $US430 million. For the individual drugs the percentage cost decrease in reimbursement amount resulting from exclusivity expiration and generic entry ranged from 24.4% for enalapril to 3.8% for pravastatin sodium.
	Kesselheim et al. 2006 [100]	Retrospective econometric study to assess the cost of market-exclusivity extensions, artificially elevated generic prices, and slow adoption of generics when therapeutically equivalent generics could have been available sooner and at lower prices.	The delay in availability, elevated prices, and slow uptake of generic alternatives for amoxicillin/clavulanate, metformin, and omeprazole alone cost Medicaid $US1.5 billion in 2000–2004.
**Patent term extensions**	**Canada**		
	The Parliamentary Budget Officer 2018 [101]	Prospective modelling study to examine the fiscal cost to the Canadian federal government of patent extension.	The increase in expenditures nationally would have reached roughly $C392 million had the two-year certificates of supplementary protection been fully in place in 2015. For provincial public programs, the cost would have been $C214 million.
	Di Matteo and Grootendorst 2002 [102]	Retrospective econometric modelling to estimate the socioeconomic and demographic determinants of real per capita provincial government drug expenditure in Canada over 1975-2000.	Any significant effects of the patent-term extensions of 1987 (Bill C-22) and 1993 (Bill C-91) on provincial drug expenditures occurred after 1995. The analysis suggests that either Bill C-91 had a more pronounced effect on drug expenditures than did Bill C-22, or the effect of the earlier legislation on drug expenditures operated with a lag, or the legislation had little effect on drug costs, and the post-1995 increase in drug expenditures is due to other factors, such as the introduction of new drugs that would have been introduced irrespective of Canada’s patent legislation
	**Australia**		
	Harris et al. 2013 [103]	Retrospective econometric study to examine the Australian provisions for extending the terms of eligible pharmaceutical patents to determine their effectiveness in securing timely access to competitively priced pharmaceuticals and in supporting innovation and employment in the industry.	The estimate of cost of patent term extensions for 2012-13 is around $A 240 million in the medium term and, in 2012 dollars, around $A 480 million in the longer term. The Review finds that the increased patent protection afforded by increasing patent life has not led to an increase in investment in Australian pharmaceutical R&D that is commensurate with the cost to Australia.
**Secondary patenting**	**USA**		
	Kapczynski et al. 2012 [104]	Retrospective study to examine the prevalence of secondary patents and their impact on patent life.	Independent formulation patents add an average of 6.5 years of patent life, independent method of use patents add 7.4 years, and independent patents on polymorphs, isomers, prodrug, ester, and/or salt claims add 6.3 years. There is evidence that late-filed independent secondary patents are more common for higher sales drugs.
	Amin and Kesselheim 2012 [19]	Retrospective study to examine how secondary patents can extend market exclusivity and thus delay generic competition. Study based on two key antiretroviral drugs: ritonavir (Norvir) and lopinavir/ritonavir (Kaletra).	108 patents were identified, which together could delay generic competition until at least 2028—12 years after the expiration of the patents on the drugs’ base compounds and 39 years after the first patents on ritonavir were filed. Some of the secondary patents that were reviewed were found to be of questionable inventiveness.
	Feldman 2018 [105]	Retrospective study to examine the proportion of Orange Book entries which indicate evergreening. Considers both patent and data exclusivity entries.	Rather than creating new medicines, pharmaceutical companies are largely recycling and repurposing old ones. Specifically, 78% of new Orange Book records were for existing drugs. Extending protection is particularly pronounced among blockbuster drugs. Once companies start down the road of extending protection, they show a tendency to do this multiple times. Increase over time in patents added. New data exclusivities focus on orphan drugs, new patient populations, new products and new uses.
	Beall et al. 2019 [106]	Retrospective study to determine whether patents on medical devices effectively extend the monopoly period for pharmaceuticals and whether this constitutes evergreening.	Unexpired device patents exist for 90% of the 49 medicine/device product combinations studied and were the only sort of unexpired patent for 14 products. Overall, 55% of the 235 patents found were device patents. Comparing the last-to-expire device patent to the last-to-expire active ingredient patent, the median additional years of patent protection afforded by device patents was 4.7 years. Incremental, patentable innovation in devices to extend the overall patent protection of medicine/device product combinations is very common.
	Yin 2015 [107]	Retrospective study to assesses the welfare gains from incremental innovation in pharmaceuticals.	Consumer surplus loss due to market exclusivity extensions far exceeds the consumer surplus benefits from incremental innovation. Market exclusivity granted for incremental innovations in SSRIs has resulted in a $US37 billion social welfare loss from 1996 to 2011. The result suggests that policy makers should reconsider the provisions for granting market exclusivity to incremental innovations.
	Hao et al. 2015 [108]	Retrospective statistical analysis to assess trends in fixed dose combinations (FDCs) and single active ingredients approved by the FDA in the period 1980-2012. Focuses on consequent time lags and estimates the effective patent and exclusivity life of FDC drugs compared to the single active ingredients included in the combination.	FDC drugs which are not new molecules added a median of 9.70 years to the patent and exclusivity life of the single active ingredients in the combination. FDC drug approvals significantly increased over the last 20 years. Pharmaceutical companies market FDC drugs shortly before the generic versions of the single ingredients enter the market extending the patent and exclusivity life of drugs now included in the combination.
	**Other countries**		
	Moir 2016 [109]	Retrospective empirical case study exploring two types of secondary ‘evergreening’ patents—new formulations and closely related chemical variants. Quantifies the cost of such evergreening patents to the Pharmaceutical Benefits Scheme.	Two evergreening venlafaxine patents led to an additional transfer of at least A$150 million from Australian taxpayers to Pfizer, beyond the transfers supported by the original new molecule patent. One was a formulation patent and the other a metabolite of the original medicine. Omeprazole patents have led to a delayed generic entry cost to taxpayers of an estimated A$1.1 billion over8 years. Over the 12-year period since the isomer, esomeprazole, came onto the market, the additional cost to the taxpayer from prescribing this higher priced molecular variant was A$1.8 billion.
	Sampat and Shadlen 2015 [110]	Retrospective comparison of the differences between primary and secondary patent grant rates in three developing countries to differences in three patent offices (the US, Europe, and Japan) that do not have measures to limit secondary patents.	Grant rates for secondary patents were lower than those for primary patents in Argentina, but not in India or Brazil. For all three countries, measures to restrict secondary patents have limited effectiveness and are not the main factor in determining grant rates – other procedural issues are more important. Includes comparison grant rates for secondary patents in developing countries for “twins,” (the same applications filed in different jurisdictions).
	Sampat and Shadlen 2017 [111]	Retrospective study to compare national approaches toward secondary pharmaceutical patents and examine outcomes of secondary patent applications in India and Brazil.	Brazil is less likely to grant applications than India, but in both countries the measures designed to limit secondary patents are having little direct effect. There may be cause for concern that laws on the books are not having the expected impact on patent outcomes in practice. At the drug level, the effects of countries’ approaches toward secondary patents needs to be understood in the context of their broader approaches toward TRIPS implementation, including when and how they introduced pharmaceutical patents in the 1990s and 2000s.

#### Data exclusivity

All three studies under this theme were from the USA. These three retrospective studies examined the impact of data exclusivity on the price of specific drugs [98], the cost to the Utah Medicaid drug program [99] and the National Medicaid drug program [100]. Kesselheim et al. found data exclusivity resulted in a delay in availability, elevated drug prices and slowed the uptake of generic alternatives which cost Medicaid $1.5 billion over 4 years [100]. These findings were reinforced by Nelson et al’s study which found a 6 month data exclusivity extension, for three classes of drugs, cost Utah’s Medicaid program $US2.2 million over 18 months, projected nationally to cost $US430 million [99]. Similarly, another study by Kesselheim and Soloman found data exclusivity extensions due to a new indication raised drug prices by a factor of more than 50 [98].

#### Patent term extensions

This theme included two studies from Canada and one from Australia. A prospective modelling study from Canada measured the cost to the Federal government of patent term extensions caused by the introduction of SPCs, a requirement of CETA [101]. This is estimated to lead to higher provincial and national drug expenditure [101]. Another study from Canada costed the impact of the introduction of the patent term extension legislative changes of 1987 and 1993 to the Canadian Patent Act and found that there was negligible impact on provincial drug prices before 1995; however patent term extension could be responsible for the very large increases incurred since then [102].^19^

The last study in this theme is the independent pharmaceutical patent review commissioned by the Australian government which was tasked with examining whether Australia’s patent system was effective in securing the timely access of competitively priced pharmaceuticals. It found that patent term extension provisions led to significantly increased costs, ($AU240 million in the short term and $AU480 million in longer term costs) [103].

#### Secondary patenting

Six of the nine articles under this theme are from the USA. Kapzynski et al. measured the prevalence of secondary patents for their impact on patent duration. They found that secondary patents were common, especially for blockbuster drugs, and that they added an additional 6-7 years to patent terms [104]. Similarly, Feldman examined the extent and regularity of evergreening behaviour in the USA over time and found that 78% of new entries in the Orange Book were for existing drugs [105]. Like Kapzynski, Feldman found that secondary patenting was particularly common among blockbuster drugs and that 80% of those who added protections added more than one [105].

A case study that investigated the number and nature of patents on two ARV medicines had similar results [19]. This identified 108 patents that could collectively delay generic competition as long as 12 years after the patent expiration of the drug’s base compounds and 39 years after the first patents on the drug. Many patents identified were of questionable validity [19]. Another study calculated the added patent duration on medical devices required to deliver medicines by examining four common conditions treated by a device/medicine combination. This found that 90% of the 49 device/medicines studied had unexpired device patents [106]. Device patents were the only unexpired patent for 14 medicines. This type of patent extended the median patent term duration on delivery of the medicine by almost 5 years [106].

A statistical modelling study measured the welfare gain from increased innovation in pharmaceuticals and found that the loss (to the community) due to extensions in data exclusivity far exceeded the gain (to originator companies) from incremental innovation or secondary patents [107]. Overall, secondary patents resulted in large social welfare losses [107]. A statistical modelling study measured the time lag between approvals for single active ingredient and fixed dose combinations (FDC) and found that FDC drug approvals had increased significantly in recent years [108]. Additionally, non-NME FDCs added a median of 9.7 years to the patent life of the single active ingredient medicines in the combination [108]. Pharmaceutical companies often market FDCs shortly before generic versions of the single active ingredients drugs are due to enter the market, extending the patent life of all drugs in the combination [108].

An Australian case study estimated the costs of delayed entry of generics for two medicines due to two types of secondary patents: new formulations and closely related chemical variants. Delayed entry of generics due to such secondary patents cost over $AU1.1 billion over 8 years for one drug and $AU150 million for another drug [109].

The final two studies by Sampat and Shadlen are country comparisons of primary and secondary patent grant rates. The first study included three developing countries (India, Brazil and Argentina) and compared them to the USA, the EU and Japan [110]. It found that measures to restrict secondary patents in developing countries have very limited impact and do not significantly determine patent grant rates. Secondary grant rates were lowest in Brazil and highest in the USA. Argentina’s measures to restrict secondary patents appear to be the most successful [110]. Sampat and Shadlen’s other study compared the effectiveness of Indian and Brazilian measures and policies to limit secondary patents [111]. Findings indicate that Brazil is less likely to grant applications than India, but in both countries, measures designed to limit secondary patents were relatively ineffective [111].

## Conflict of interest

Several studies disclosed funding from research-based pharmaceutical companies [62, 63] and associations [64, 88, 89] and one from a generic and biosimilars association [57]. Of these, the aforementioned Djolov and Rozek articles’ [88, 89] findings differed from the rest of the articles and scored poorly on our quality assessment scale. These studies had little to no impact on the predominant findings of the review given their low-quality scores and contrary findings to the other 89 included studies. Other industry funded studies [57, 62–64] used sound methodologies (according to our quality assessment scale) and had findings consistent with the vast majority of studies.

This systematic review included a small number of papers that were authored by some of this review’s authors [35, 74, 109]. Quality assessment of these articles was delegated to a team member who was not an author. None of these papers had findings that deviated from the predominant findings of the review.

## Quality of articles

A small number of studies scored lower on our quality assessment scale due to a lack of detail in the methodology [26] or methodological flaws [63] and assumptions. Some relied on modelling that did not measure variables at appropriate points in time [34, 79] or demonstrate the existence of a correlation or causal relationship between policy changes and indicators of access to medicines [33, 43].

## Scope

Overall, only about a third of the studies included resource poor settings, signalling a need for greater emphasis on the impact of IP laws in low and LMIC contexts where longer patent terms are likely to have more damaging impacts on access to medicines for the most vulnerable. This is particularly pertinent as some low-income countries are poised to graduate from LDC status and will no longer be exempt from granting pharmaceutical product patents.

## Discussion

The main finding of this review is that TRIPs-plus IP rules are associated with increased drug prices, consumer welfare losses and increased costs to consumers and governments.

### Trade agreements

Although more extensive IP provisions and longer monopoly periods are largely being driven by trade negotiations, most studies did not primarily examine trade agreements. Those that did, focused on US and EU trade agreements. This is not surprising given these jurisdictions are net exporters of medicines and therefore the most likely to seek TRIPS-plus IP provisions in trade agreements. The EU trade agreements studied included CETA and the EU-Andean FTA. The IP provisions in these EU trade agreements were found to have potential negative implications for access to medicines in Canada [22–24], Peru [37] and Columbia [38] by delaying the entry of generic competition and increasing pharmaceutical expenditure.

The US trade agreements studied spanned a broader range of countries. Some of the studies included regional trade agreements that included the USA, however their findings were confined to the impact of the IP provisions on a specific country. This included the impact on Thailand [25–27], Jordan [28, 39], Korea [29, 30], Canada [31, 32], Chile [34], Guatemala [33], and Vietnam [35]. All of these studies found negative implications for access to medicines in the selected country. These negative implications included increased treatment program costs and decreased treatment coverage [35], increases in drug prices [26, 27, 32, 34, 39], restricted and or delayed access to generic [26–28, 33, 39] and biosimilar drugs [32], lost savings [31], consumer [25, 29] and total welfare losses [29], and increased pharmaceutical expenditure [26, 28]. Additionally Son found that the Korea-US trade agreement failed to increase the availability of new medicines in Korea and did not shorten the drug availability lag for new medicines [30].

Of the 18 studies that focused primarily on trade agreements, the findings of one deviated significantly from the rest. Bollyky focused on the impact on all countries with US trade agreements and found no increases in the average off-patent originator drug price, pharmaceutical spending, consumption of pharmaceuticals, the proportion of branded versus generic drugs or in the national spending on pharmaceuticals as a share of total health expenditure [36]. Kapczynski et al. criticised Bollyky’s analysis as “misleading because it fails to look at the right drugs at the right points in time, overlooks the temporal dimensions of implementation of provisions in previous trade agreements, and ignores the broader context in which trade agreements are negotiated and implemented” [112]. They suggest more empirical work is needed to better understand the impact of more extensive IP provisions on access to medicines in low income countries [112]. Of relevance here is Shadlen et al’s observations that IP provisions in trade agreements come into effect at different times, making analysis complicated [10]. Many countries with trade agreements introduced pharmaceutical product patents only after TRIPS (1995). Given 20-year patent terms, it is premature to assess the impacts of TRIPS-plus provisions until patent expiry in 2015 or later for countries taking full advantage of transition periods [10].

Fifteen of the 18 studies on trade agreements were prospective modelling studies drawing on leaked text to assess the potential impacts of the agreements before they were signed. Islam et al. describe in detail the advantages and disadvantages of prospective versus retrospective analyses of trade agreement impacts [16]. They claim that while both types of studies generally find that trade agreements result in price increases and consumer welfare decreases, there is generally a difference in the size of the impact with retrospective studies tending to be more modest in their impact estimates [16]. This was attributed to assumptions made in ex-ante models and the limitations of ex-post studies [16].

### Patent policies

Several comparative country studies that measured the impact of patent regimes on time to drug launch found that more stringent patent provisions accelerate the market entry of new drugs, [81–83, 85, 94] especially ‘blockbuster’ drugs [82, 94]. Some of these studies found this phenomenon to be true only for high income countries and not low- and middle-income countries [82, 83]. This could reflect simple profit maximising behaviour, focusing on more lucrative markets and more lucrative drugs. Conversely, two studies found that higher levels of IP protection were associated with lower access to medicines [85, 87]. Even in studies that showed a positive association between longer IP protection and new drug availability, the high price of the patented pharmaceutical product resulted in a net effect of significantly reduced sales [85]. Hellerstein also found that countries with largely originator drug markets were paying significantly more for ARV than generic supply countries [86]. Relevant here is an important finding of studies on generic entry - this results in significant price falls only when there is competition among generics [113].

The findings of two comparative patent studies contrasted markedly with the studies above. Rozek and Berkowitz find that ‘improving’ IP regimes does not change the prices of branded pharmaceutical products and that countries with ‘improved’ IP regimes do not have higher priced drugs than countries without [88]. ‘Improved’ IP in this instance was equated with stronger protection of IP, but the exact meaning of this was not defined. Further, there were inadequate controls for other relevant variables [88]. Djolov also found that strong patent protection was associated with better access to essential drugs [89]. In the Djolov study [89] the essential drugs are almost all off-patent and therefore unaffected by the strength of the patent provisions. The time period studied was not long enough to capture the impact of new patent provisions [89]. To more accurately measure the relationship between patent and prices, only new previously unpatented drugs introduced after the new product patent regime should be included in the analysis [114, 115].

The nine single-country studies that focused on the impact of stronger patents all found negative implications for access to medicines. Patents were found to cause significant welfare loss [90, 91, 93] and a rise in drug prices in India [92]. Supporting these findings, the availability of generics and me-too drugs led to consumer welfare gains [80].

The studies focused on India are particularly pertinent to understanding the impact of introducing patents for pharmaceutical products. India took full advantage of the 10-year transition period to introduce such patents [116]. Further, India designed its patent legislation to ensure that closely similar medical compounds could not be patented unless there is a new therapeutic benefit. Prior to this, Indian manufacturers were legally producing generic versions of many medicines under patent in other countries. The Indian patent regime has implications for access to medicines not only in India, but around the world as it may inhibit the production of generic medicines. India is the world’s biggest producer of generic medicines and accounts for more than 80% of ARV annual purchase volumes [117]. Although these studies focused on the Indian market only, pharmaceutical product patents in India could potentially curtail generic drug supply for many of the world’s poorest countries.

### TRIPS-plus rules - patent term extensions and secondary patents

The articles that measured the impact of TRIPS-plus rules focused on patent term extensions and secondary patenting. Ten of the 13 TRIPS-plus studies were from North America, two were from Australia and one was a multi-country study that included the USA.

All patent term extension studies found negative impacts for access to medicines which included increased government health care expenditure [99–103], increased drug prices [98, 100] and delays in drug availability [100].

Similarly, all articles examining secondary patents found negative impacts for access to medicines including longer monopoly periods that delayed the market entry of cheaper generics [19, 104, 106, 107], increased costs to taxpayers [109] and social welfare losses [107]. The only article that included a focus on resource poor settings found that measures to restrict secondary patenting had limited effectiveness in Argentina and no measurable impact in India and Brazil [110]. The authors suggest that administrative and procedural features of patent systems may be a greater influence on the grant of secondary patents than patent laws themselves [110]. Tackling this issue would mean addressing the kind of technical assistance provided to patent offices in lower- and middle-income countries [118]. This is a priority issue given the expense of challenging granted patents and the impact that longer patent durations could have on access to medicines in countries less able to pay high prices for medicines. Secondary patenting in India could have negative ramifications for countries that depend on India for affordable generic drugs.

### Generic entry/patent expiry

Generic drugs are more affordable than patented drugs as they are usually more closely priced to the marginal cost of production when there are three or more generic drugs available [113]. It is therefore unsurprising to find that all articles that explored various facets of generic market entry and or patent expiry found positive impacts for access to medicines. For example, the most salient finding in this group of 22 articles was that generic market entry resulted in significant reductions in the cost of medicines [23, 58, 60, 61, 64–67, 69, 73, 75–78, 119] but not necessarily in the price of the originator drug [72]. One study found generic status to be the most consistent predictor of a drug’s price [61].

The introduction of the US Hatch-Waxman Act in 1984 was designed, inter alia, to promote early and efficient access to generic drugs. This was to be done by facilitating accelerated approval by the Food and Drug Administration (FDA), the national regulator, to allow for market entry of generics as soon as patents expired, or were otherwise found to be invalid or not infringed [14]. Studies which focused on generic market entry in the USA found that the introduction of the Hatch-Waxman Act resulted in a rise in patent challenges [63], an increase in the number of patents associated with each medicine [105] a rise in the generic share of the market [64], consumer welfare gains [62] and a decrease in daily treatment costs [64]. To maximise profits, however, many originator brands patent reformulations to lessen the financial impact of patent expiry [68]. Among best-selling drugs, Feldman found that over 70% of medicines listed in the Orange Book added additional patents or data exclusivity privileges to the medicine, indicating extensive use of secondary patenting [105].

Nearly all of these studies were set in high-income countries, however, even between EU Member States significant wealth disparities exist and access to medicines is not uniform. One multi-country EU study found that affordability constraints impacted on drug access and when generic alternatives entered the market access to effective medicines improved, most notably in lower-income EU Member States [57]. These findings have implications for settings where patents and high drug prices compromise people’s access to affordable generics.

### Compulsory licensing, parallel trade and other flexibilities

TRIPS flexibilities such as compulsory licensing or even the threat of compulsory licensing, increase drug availability [48], increase treatment coverage and decrease drug prices [48, 49]. Compulsory licensing has most commonly been implemented to facilitate greater access to ARV medication [48–50]. Interestingly, compulsory licensing seems to have become less frequent since 2006. Parallel trade appears to have a more modest impact on drug prices [52, 53] than compulsory licensing, however, there were very few studies on parallel trade, all of which are focused on EU Member States. Despite this, most showed cost savings from parallel trade [54–56]. These findings are in keeping with what TRIPS flexibilities were originally designed to do: provide governments with the ‘policy space’ to mitigate the negative impacts of patents, such as excessively high drug prices and lack of competition [120].

## Other systematic reviews

The findings of this study provide rich empirical detail and findings that add to other systematic reviews. Barlow et al’s systematic review of the health impact of trade and investment agreements, also found one study [35] showing increased access to medicines due to compulsory licensing. Islam et al’s review of IP provisions in trade treaties on access to medicines in LMICs also found that IP provisions in trade agreements increased drug prices and decreased consumer welfare [16]. However, this review did not include high income countries or IP rules unrelated to trade agreements. Trachtenberg et al’s policy brief on trade agreements and access to medicines had a narrower scope of review but included a useful critique of the methods the studies employed [121]. This review included some of the same articles and reached the same conclusions as the current study; IP provisions in trade agreements have a negative impact on the price and availability of medicines. The current review included a broader set of studies and is useful in providing a more complete assessment of the current state of knowledge.

## Limitations

This review included only English-language articles, so it is possible we excluded otherwise valid studies based on this criterion. Many included articles were focused on European and North American countries and there is a dearth of literature on resource-poor settings so we cannot draw conclusions about the effects of changes in IP in these countries. Additionally, it was very challenging to systematically scan the grey literature for papers that met the inclusion criteria due to the sheer volume of irrelevant material it generated, so relevant grey literature may have been missed.

## Conclusions

The papers in this review span various disciplines including economics, public health and social sciences. Much of the evidence comes from studies focused not on the impact of specific trade agreements, but on specific TRIPS-plus provisions implemented independently of trade agreements.

With very few exceptions, the articles in this review found that the introduction of patents following implementation of TRIPS rules and pharmaceutical-related TRIPS-plus IP provisions are associated with an increase in drug prices, consumer welfare losses and increased costs to consumers of pharmaceuticals and national governments. Studies in this field can be complex and the quality of the findings is dependent on when impacts are measured, the context and choice of methods.

There is evidence that TRIPS flexibilities can facilitate access to medicines although their use is limited and their implementation sometimes challenging – they cannot alone be a panacea to IP provisions that increase monopolies. They can, however, offer countries some flexibility and negotiating leverage with pharmaceutical companies in determining their access and pricing with regards to national markets. The same is true of some country-based policies designed to increase access to affordable generic medicines. IP laws do not operate outside social, political and economic contexts whether at local or global levels. In particular, they interact with a range of other laws that are likely to impact access to medicines in a given jurisdiction, which although outside the scope of this paper, merits further research. There is clearly also a need for further research to advance the policy-oriented research agenda developed in 2019 by the Working Group on Trade, Investment Treaties and Access to Medicines, particularly to strengthen quantitative studies of the impact of treaties on access to medicines for people in LMICs [122].

## Supplementary Information

**Additional file 1.** Systematic review data extraction form.

**Additional file 2.** Quality assessment form.

## Data Availability

The datasets used and/or analysed during the current study are available from the corresponding author on reasonable request.

## Abbreviations

ARV: Anti-retroviral (medication)
EU: European Union
CAFTA: Central America free trade agreement
CETA: Comprehensive Economic and Trade Agreement
CUSMA/USMCA/T-MEC: Canada-United States-Mexico Agreement/ United States–Mexico–Canada Agreement/ Tratado entre México, Estados Unidos y Canadá
FDC: Fixed dose combinations
FTA: Free trade agreement
IP: Intellectual property
LDC: Least developed country
LMIC: Lower-middle-income country
NME: New molecular entities
PBS: Pharmaceutical Benefits Scheme
PTE: Patent term extension
SPC: Supplementary Protection Certificates
TPP: Trans-Pacific Partnership
TRIPS: Trade Related Aspects of Intellectual Property Rights Agreement
US / USA: United States (of America)
WTO: World Trade Organization

## Footnotes

1 We use the term ‘access’ is this review to mean the medicine is available at an affordable price.

2 Cost refers to the expense incurred by governments and insurance schemes and includes economic measures such as welfare costs. In contrast, price refers to what is charged for a drug.

3 Some articles included components that did not meet our inclusion criteria and hence those findings were not included in our review.

4 Prospective and retrospective refer to the timing of the research in relation to the data used in the study. In retrospective studies, the data are in respect of past years. Prospective studies use modelling or statistical analysis to estimate a likely future outcome.

5 Supplementary Protection Certificates are the EU system for patent term extensions, granted due to delays in marketing approval (https://e-courses.epo.org/wbts_int/litigation/SPCs.pdf).

6 Consumer welfare refers to the individual benefits derived from the consumption of goods and services (https://stats.oecd.org/glossary/detail.asp?ID=3177).

7 This is also known as ‘USMCA’ in the US and ‘T-MEC’ in Mexico. It supersedes the 1994 North American Trade Agreement (NAFTA).

8 The article is based on leaked 2014 text from the TPP IP chapter before the USA withdrew and it became the CPTPP – hence its inclusion in the ‘FTAs that include US’ section.

9 Consumers’ surplus is a measure of consumer welfare and is defined as the excess of social valuation of product over the price actually paid (https://stats.oecd.org/glossary/detail.asp?ID=3176).

10 And all focused on the impact in the receiving countries. None of the included studies looked at the impacts in the exporting countries.

11 In 1984, the US Congress passed The Drug Price Competition and Patent Term Restoration Act generally known as the “Hatch-Waxman Act.” The Hatch-Waxman rules created processes and incentives for both branded and generic companies [11]. Data exclusivity provisions favour originator companies, while abbreviated approval processes and rewards for successfully challenging weak patents (Paragraph IV) reward generic companies.

12 This study also looked a pay-for-delay settlements, where originator companies pay generic companies not to challenge weak patents. Such articles were excluded from the scope of the study – they are found only in the USA and Europe and in recent years courts have issued substantial fines for such behaviour.

13 A seminar by Jayashree Watal (at http://www.wipo.int/edocs/mdocs/scp/en/scp_21/scp_21_ref_watal.pdf) on Bolar provisions refers to a 1998 US Congressional Budget Office paper (at https://www.cbo.gov/sites/default/files/105th-congress-1997-1998/reports/pharm.pdf) which estimates that following the Hatch-Waxman Act, the average delay between first patent expiry and generic entry fell from more than 3 years to less than 6 months. This paper falls outside the terms of our review but confirms the findings of studies included in the review.

14 A blockbuster drug is one with a very high global sales revenue – the first such drug (cimetidine (Tagamet)) achieved revenues in excess of US$1 billion (late 1970s). Since then the revenue threshold for blockbusters has increased to annual global revenues of US$2.5 billion or more. Other examples of blockbuster drugs are Lipitor, Losec and Humira.

15 Reference [80], discussed under patent term extensions (PTEs) also concerns the Canadian legislation on PTEs. PTEs delay generic entry because of the extension in the patent monopoly period.

16 As did Moir et al. [32] discussed above under trade agreements.

17 The Ginarte Park index claims to measure IP ‘strength’ but is criticised simply as a measure of applicant friendliness

18 A *me-too drug* is a compound that is structurally related to a first-in-class compound, belongs to the same therapeutic class and is used for the same therapeutic purposes, but which differs in some respects, such as adverse reactions profile, or drug–drug interactions.

19 In 1987 the Canadian Patent Act was amended to delay permitting compulsory licenses. It also extended patent duration from 7 to 10 years. It was further amended in 1993 to exclude compulsory licencing and extend patent duration to 20 years.
