# Supplementary material for: What is the impact of intellectual property rules on access to medicines? A systematic review
Source: Global Health. 2022 Apr 15;18:40. doi: 10.1186/s12992-022-00826-4 (PMC9013034; doi:10.1186/s12992-022-00826-4)
Supplement: Supplementary file 1 — Additional file 1. Systematic review data extraction form. [file 12992_2022_826_MOESM1_ESM.docx]

**Supplementary file 1: Systematic review data extraction form**

**The impact of intellectual property settings on the cost, price and availability of medicines**

| Title: | | | Assessor: |
| --- | --- | --- | --- |
| Author(s): | | Covidence |  |
| Publication date: |  | Date assessed: |  |
| Years studied: |  | | |
| Country / countries |  | | |
| Research question/ objective: |  | | |
| Study design: |  | | |
| Study data |  | | |
| IP setting(s) studied: |  | | |
| Outcome measure(s): |  | | |
| Method of analysis: |  | | |
| Main findings: |  | | |
| Funding source: |  | | |
| Notes/comments: |  | | |
