# Supplementary material for: What is the impact of intellectual property rules on access to medicines? A systematic review
Source: Global Health. 2022 Apr 15;18:40. doi: 10.1186/s12992-022-00826-4 (PMC9013034; doi:10.1186/s12992-022-00826-4)
Supplement: Supplementary file 2 — Additional file 2. Quality assessment form. [file 12992_2022_826_MOESM2_ESM.docx]

**Supplementary file 2: Quality assessment form**

**The impact of intellectual property settings on the cost, price and availability of medicines**

| Title: | | | | | | | Assessor: |
| --- | --- | --- | --- | --- | --- | --- | --- |
| Author(s): | | | | | Covidence # | |  |
| Publication date: |  | | | | Date assessed: | |  |
|  | Good | Fair | Poor | Very poor | | Comment | |
| 1. Abstract and title |  |  |  |  | |  | |
| 2. Introduction and aims |  |  |  |  | |  | |
| 3. Method and data |  |  |  |  | |  | |
| 4. Sampling |  |  |  |  | |  | |
| 5. Data analysis |  |  |  |  | |  | |
| 6. Ethics and bias |  |  |  |  | |  | |
| 7. Findings/results |  |  |  |  | |  | |
| 8. Transferability/ generalisability |  |  |  |  | |  | |
| 9. Implications and usefulness |  |  |  |  | |  | |
| Total |  |  |  |  | |  | |
| Notes/comments: |  | | | | | | |
